# Transcriptional programming of lipid and amino acid metabolism by the skeletal muscle circadian clock

**DOI:** 10.1371/journal.pbio.2005886

**Published:** 2018-08-10

**Authors:** Kenneth Allen Dyar, Michaël Jean Hubert, Ashfaq Ali Mir, Stefano Ciciliot, Dominik Lutter, Franziska Greulich, Fabiana Quagliarini, Maximilian Kleinert, Katrin Fischer, Thomas Oliver Eichmann, Lauren Emily Wright, Marcia Ivonne Peña Paz, Alberto Casarin, Vanessa Pertegato, Vanina Romanello, Mattia Albiero, Sara Mazzucco, Rosario Rizzuto, Leonardo Salviati, Gianni Biolo, Bert Blaauw, Stefano Schiaffino, N. Henriette Uhlenhaut

**Affiliations:** 1 Helmholtz Diabetes Center (HMGU) and German Center for Diabetes Research (DZD), Institute for Diabetes and Obesity (IDO), Munich, Germany; 2 Venetian Institute of Molecular Medicine (VIMM), Padova, Italy; 3 Institute of Molecular Biosciences, University of Graz, Graz, Austria; 4 Department of Biomedical Sciences, University of Padova, Padova, Italy; 5 Clinical Genetics Unit, Department of Woman and Child Health, University of Padova, and IRP Città della Speranza, Padova, Italy; 6 Clinica Medica, Department of Medical Sciences, University of Trieste, Trieste, Italy; 7 Gene Center, Ludwig-Maximilians-Universitaet (LMU), Munich, Germany; Duke University, United States of America

## Abstract

Circadian clocks are fundamental physiological regulators of energy homeostasis, but direct transcriptional targets of the muscle clock machinery are unknown. To understand how the muscle clock directs rhythmic metabolism, we determined genome-wide binding of the master clock regulators brain and muscle ARNT-like protein 1 (BMAL1) and REV-ERBα in murine muscles. Integrating occupancy with 24-hr gene expression and metabolomics after muscle-specific loss of BMAL1 and REV-ERBα, here we unravel novel molecular mechanisms connecting muscle clock function to daily cycles of lipid and protein metabolism. Validating BMAL1 and REV-ERBα targets using luciferase assays and in vivo rescue, we demonstrate how a major role of the muscle clock is to promote diurnal cycles of neutral lipid storage while coordinately inhibiting lipid and protein catabolism prior to awakening. This occurs by BMAL1-dependent activation of *Dgat2* and REV-ERBα-dependent repression of major targets involved in lipid metabolism and protein turnover (*MuRF-1*, *Atrogin-1*). Accordingly, muscle-specific loss of BMAL1 is associated with metabolic inefficiency, impaired muscle triglyceride biosynthesis, and accumulation of bioactive lipids and amino acids. Taken together, our data provide a comprehensive overview of how genomic binding of BMAL1 and REV-ERBα is related to temporal changes in gene expression and metabolite fluctuations.

## Author summary

Circadian clocks are known to regulate local and systemic homeostasis by anticipating rhythmic changes in behavior and nutritional state and by compartmentalizing incompatible metabolic pathways within precise temporal and spatial windows. Yet a precise mechanistic understanding of how the circadian clock in skeletal muscle controls homeostasis is just beginning to come to light. Here, we investigated how the muscle clock directs 24-hr metabolic rhythms. We compared genome-wide binding of clock transcription factors brain and muscle ARNT-like protein 1 (BMAL1) and REV-ERBα with 24-hr transcriptional and metabolic effects after their loss of function specifically in muscles. We found that the muscle clock plays a major role anticipating the transition from fasting to feeding. This occurs by direct activation of transcriptional programs promoting lipid storage, insulin sensitivity, and glucose metabolism, with coordinated repression of programs controlling lipid oxidation and protein catabolism. Importantly, these gene expression changes occur in the hours prior to systemic metabolic and hormonal cues that arise upon awakening. As such, we find that the muscle clock tips the scales in favor of glucose metabolism, whereas loss of function of the clock transcription factor BMAL1 is associated with persistent lipid metabolism, protein catabolism, and metabolic inefficiency.

## Introduction

Circadian rhythms of metabolism are endogenously generated and maintained by tissue-specific gene networks under the transcriptional control of molecular clocks [1]. While chronic misalignment/disruption of circadian clocks has consistently been linked to metabolic disorders and diseases [2], precise pathogenic mechanisms and their relation to tissue-specific clock function remain largely undefined.

Global and tissue-specific conditional or inducible loss-of-function mouse models targeting clock genes have begun to unravel such relationships in skeletal muscle [3], a multifaceted and highly dynamic tissue and a major player in whole-body energy homeostasis. Depending on fluctuations in energy supply and demand, skeletal muscle plays various essential metabolic roles in the uptake, storage, utilization, and release of oxidative substrates. Experimental efforts have established muscle as the main site for insulin-stimulated glucose disposal [4] and a major consumer of lipoprotein-triacylglycerol-derived fatty acid and plasma free fatty acids (FFAs) [5]. Importantly, muscle tissue serves a highly dynamic role as the main destination for circulating amino acids in the fed state and the main source of circulating amino acids during starvation and insulin deficiency [6].

Using muscle-specific knockout (myocyte-specific loss of BMAL1 [mKO]) models of *Bmal1*, an essential and nonredundant core clock transcriptional activator, we previously demonstrated that a critical function of the muscle clock is to anticipate and promote diurnal changes in glucose uptake and oxidation prior to the sleep–wake transition [7]. Accordingly, loss of brain and muscle ARNT-like protein 1 (BMAL1) is associated with reduced insulin sensitivity and glucose oxidation. However, no previous study has systematically addressed the muscle clock’s specific role in lipid and amino acid metabolism or the impact of muscle-specific *Bmal1* deletion on whole-body energy homeostasis. Furthermore, direct transcriptional targets of clock transcription factors in skeletal muscle remain mostly unknown.

BMAL1 forms a heterodimeric transcriptional activator together with circadian locomotor output cycles kaput (CLOCK) and constitutes an integral component of the core molecular oscillator [8]. Global loss of BMAL1 results in loss of circadian physiology [9], impaired entrainment of circadian behaviors to light/dark cycles [10], and loss of rhythmic expression of canonical BMAL1 targets. These include *Rev-erbα (Nr1d1)* and *Rev-erbβ (Nr1d2)* [11], each coding for dominant transcriptional repressors. REV-ERBα/β repress their targets by competitively binding DNA response elements recognized by other nuclear receptors, especially the RAR-related orphan receptor (ROR) constitutive activators, but also by recruiting the nuclear receptor corepressor 1–histone deacetylase 3 complex (NCoR1-HDAC3) [12] and by indirectly binding to tissue-specific transcription factors [13]. Circadian accumulation of REV-ERBα/β likewise causes rhythmic repression of target genes, including *Bmal1*. Together, BMAL1 and REV-ERBα thus form important positive and negative elements of the circadian clock.

To identify additional metabolic roles of the muscle clock, we created and integrated multiple in vivo high-throughput “omics” datasets. Here, we present a comprehensive in vivo map of BMAL1 and REV-ERBα genomic binding in adult mouse skeletal muscle and highlight specific transcriptional and metabolic consequences of muscle-specific loss of BMAL1 and REV-ERBα. We determine how BMAL1 binding can activate expression of *Dgat2*, thus promoting diurnal cycles of neutral lipid storage. We also show how REV-ERBα can coordinately inhibit a network of master regulators of lipid and protein metabolism, thus tempering diurnal rhythms of lipid oxidation and physiological protein turnover. Importantly, these changes occur prior to and independent of metabolic and hormonal cues that arise during the feeding phase, i.e., when glucose becomes the predominant fuel source. In addition, we show how loss of BMAL1 is associated with disruption of its target genes, including *Rev-erbα*, and leads to a state of metabolic inefficiency characterized by impaired neutral lipid storage, increased lipid catabolism and oxidation, increased muscle protein turnover, mild mitochondrial uncoupling, and increased energy expenditure (EE). Overall, our data bring to light previously uncharacterized molecular circuits underlying metabolic efficiency within skeletal muscle and illustrate mechanistically how circadian transcription factors can anticipate and confine the use of energy substrates to distinct temporal windows.

## Results

### Genome-wide binding of clock transcription factors BMAL1 and REV-ERBα in mouse skeletal muscle reveals tight coordination between factors

To understand how the muscle clock can transcriptionally direct rhythmic metabolism, we mapped in vivo genome-wide chromatin occupancy (cistromes) of the positive master clock regulator BMAL1 and the dominant repressor REV-ERBα in adult mouse gastrocnemius muscles. Accordingly, we performed chromatin immunoprecipitation followed by next-generation sequencing (ChIP-seq) on tissues collected during maximum diurnal protein expression for BMAL1 and REV-ERBα at Zeitgeber time (ZT) 4 and 8, respectively (S1A Fig).

**Fig 1:**
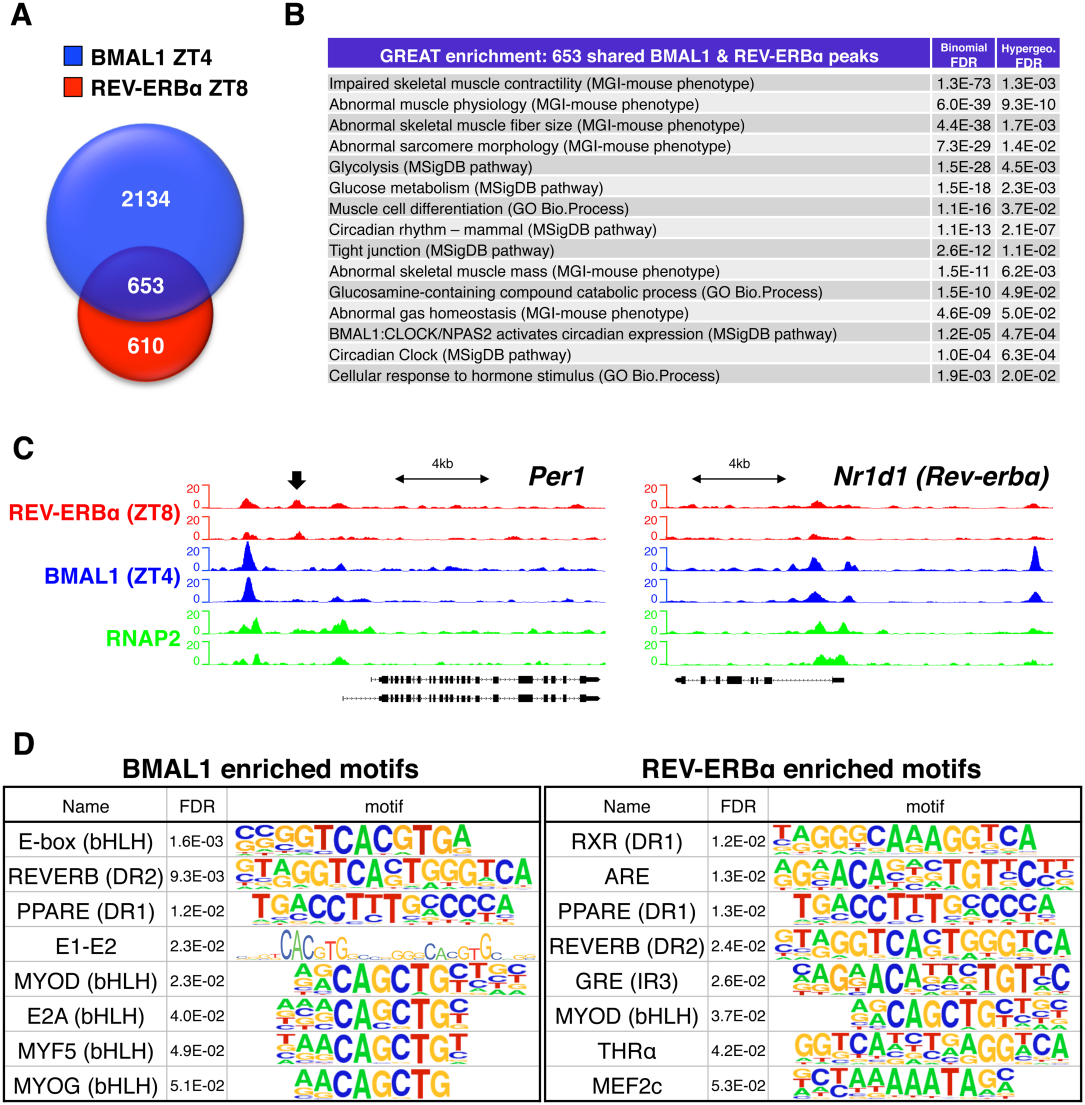
Genome-wide binding of clock transcription factors BMAL1 and REV-ERBα in mouse skeletal muscle. (A) Overlap of BMAL1 and REV-ERBα “high confidence” peaks identified in gastrocnemius muscle from WT mice. (B) Functional enrichment analysis (GREAT) of 653 shared muscle BMAL1 and REV-ERBα peaks. (C) Aligned genome browser tracks showing binding of BMAL1, REV-ERBα, and RNAP2 at selected clock-associated transcriptional regulators. The left axis indicates sequence-tag counts. Arrow indicates a REV-ERBα-specific peak in the *Per1* promoter. (D) Representative top-ranking motifs found in chromatin sites occupied by BMAL1 and REV-ERBα in vivo (selected from S2 Table). Underlying data can be found in supporting files S1 and S2 Tables, and at Gene Expression Omnibus (accession number GSE108650). ARE, androgen response element; bHLH, basic helix-loop-helix; BMAL1, brain and muscle ARNT-like protein 1; CLOCK, circadian locomotor output cycles kaput; DR1, direct repeat 1; DR2, direct repeat 2; FDR, false discovery rate; GO, Gene Ontology; GRE, glucocorticoid response element; GREAT, Genomic Regions Enrichment of Annotations Tool; IR3, inverted repeat 3; MEF2, myocyte enhancer binding factor 2; MGI, Mouse Genome Informatics; MSigDB, Molecular Signatures Database; MYF5, myogenic factor 5; MYOD, myoblast determination protein; MYOG, myogenin; NPAS2, neuronal PAS domain protein 2; PPARE, peroxisome proliferator–activated receptor response element; RNAP2, RNA polymerase II; RXR, retinoid X receptor; THRα, thyroid hormone receptor alpha; WT, wild type; ZT, Zeitgeber time.

We identified 2,787 BMAL1 and 1,263 REV-ERBα “high confidence” peaks (i.e., reproducible ChIP-seq peaks from 2 biological replicates), with 653 peaks showing “confident” occupancy by both factors (i.e., peaks shared by ≥3 of the 4 samples) (Fig 1A and S1 Table). Extensive overlap between muscle BMAL1 and REV-ERBα targets is in agreement with previous reports for mouse liver [14,15], suggesting that BMAL1 and REV-ERBα occupy many of the same *cis*-regulatory genomic sites and regulate many of the same targets, albeit at different times. Functional enrichment analysis [16] of these shared genomic sites underscored their shared regulation of circadian processes, as well as their maintenance of myofiber form and function (Fig 1B). For example, in addition to shared roles regulating the “Circadian Clock” and “Circadian rhythm” pathways, common muscle BMAL1 and REV-ERBα peaks were associated with genes regulating muscle mass and contractility, muscle cell differentiation, glucose and glucosamine metabolism, and the cellular response to hormones.

Consistent with their integral role regulating core clock gene expression, we identified highly enriched common muscle BMAL1 and REV-ERBα peaks at promoters and enhancers of clock-associated transcriptional regulators like *Per1*, *Per2*, *Cry1*, *Cry2*, *Rev-erbα* (*Nr1d1*), *Rev-erbβ* (*Nr1d2*), *Dbp*, *Tef*, *Dec1* (*Bhlhe40*), *Dec2* (*Bhlhe41*), and *Chrono* (*Ciart/Gm129*) (Fig 1C and S1C Fig). We also noted common BMAL1 and REV-ERBα peaks near several known [17] muscle clock-dependent circadian genes, including *Coq10b*, *Dgat2*, *Klf9*, *Mylk4*, *Tcap*, and *polybubiquitin-C* (*Ubc*) (S1D Fig).

Comparing our muscle cistromes to published mouse liver cistrome data for BMAL1 [14] and REV-ERBα [15], we found only 46 common BMAL1 peaks between tissues, associated with 42 common genes, and 264 common REV-ERBα peaks associated with 252 common genes (S1B Fig and S1 Table). Common muscle and liver targets were mainly core circadian clock and known clock-dependent output genes. Interestingly, common muscle and liver REV-ERBα targets were additionally enriched for p53 signaling components, chromatin modifiers, as well as macroautophagy and mitophagy mediators. However, the vast majority of sites (98% of BMAL1 peaks and 80% of REV-ERBα peaks) were muscle specific. This implies highly tissue-specific roles for BMAL1 and REV-ERBα beyond their common regulation of core clock genes.

To verify specificity of our muscle cistrome data and uncover potentially novel muscle-specific transcriptional networks, we searched BMAL1 and REV-ERBα peaks for known transcription factor motifs. In agreement with their high degree of functional overlap, we found significant enrichment for E-boxes and the canonical REV-ERB direct repeat 2 (DR2) motif among the top-ranking motifs of each factor (Fig 1D, S2A–S2E Fig, S2 Table and S2 Data). Both factors also showed a muscle-specific genomic signature, with enrichment for the basic helix-loop-helix (bHLH) myogenic regulatory factors myoblast determination protein (MYOD), myogenic factor 5 (MYF5), and myogenin (MYOG), in addition to multiple isoforms of their coregulator, the myocyte enhancer binding factor 2 (MEF2) [18]. Highlighting potential loci for cross-talk between circadian clock components and hormones [19], both BMAL1 and REV-ERBα peaks also showed motif enrichment for several different nuclear hormone receptors with relatively high diurnal expression levels in adult skeletal muscle (S3A Fig), including androgen receptor (AR; androgen response element [“ARE”]), retinoid X receptor (“RXR”), peroxisome proliferator–activated receptor (PPAR; PPAR response element [“PPARE”]), glucocorticoid receptor (GR; glucocorticoid response element [“GRE”]), and thyroid hormone receptor (TR) alpha (“THRα”). In summary, our in vivo cistromes indicate that muscle BMAL1 and REV-ERBα sequentially bind and regulate many of the same target genes, yet in a decisively muscle-specific manner.

### Transcriptional reprogramming of metabolism after muscle-specific loss of BMAL1

**Fig 2:**
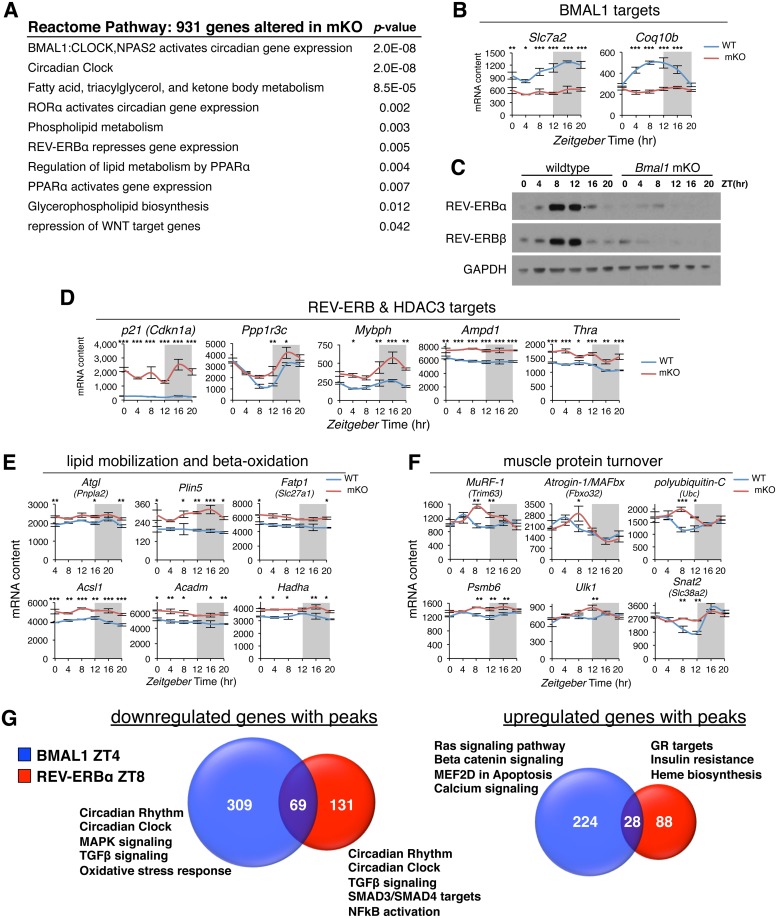
Transcriptional reprogramming of metabolic pathways in mKO muscles. (A) REACTOME pathway enrichment analysis of 931 differentially expressed genes from mKO TA muscles. (B) Diurnal expression profiles of selected BMAL1-dependent circadian genes in TA determined by microarray and plotted as absolute expression levels (*n* = 3 × timepoint; mean ± SEM *p = 0.05, **p = 0.01, ***p = 0.001, 2-way ANOVA with Bonferroni correction). (C) Diurnal REV-ERBα and REV-ERBβ protein levels determined by western blot in vastus lateralis; GAPDH used as loading control. (D-F) Diurnal expression profiles in TA muscle of selected (D) REV-ERBα and HDAC3 target genes, (E) PPARα/δ-regulated mediators of lipid catabolism and oxidation, (F) GR-regulated mediators of protein turnover (*n* = 3 × timepoint; mean ± SEM *p = 0.05, **p = 0.01, ***p = 0.001, 2-way ANOVA with Bonferroni correction). (G) Venn diagram showing relative overlap and pathway enrichment of differentially regulated mKO genes with BMAL1 and REV-ERBα ChIP-seq peaks. Underlying data can be found in supporting files S1 Data, S1 Table, and at Gene Expression Omnibus (accession number GSE43071). BMAL1, brain and muscle ARNT-like protein 1; ChIP-seq, chromatin immunoprecipitation followed by next-generation sequencing; CLOCK, circadian locomotor output cycles kaput; GAPDH, glyceraldehyde 3-phosphate dehydrogenase; GR, glucocorticoid receptor; HDAC3, histone deacetylase 3; MAPK, mitogen-activated protein kinase; MEF2, myocyte enhancer binding factor 2; mKO, myocyte-specific loss of BMAL1; NFκB, nuclear factor kappa B; NPAS2, neuronal PAS domain protein 2; PPARα/δ, peroxisome proliferator–activated receptor alpha/delta; RORα, RAR-related orphan receptor alpha; TA, tibialis anterior; TGFβ, transforming growth factor beta; WT, wild type; ZT, Zeitgeber time.

To provide a functional context for associations between BMAL1 and REV-ERBα binding sites and target gene regulation, we focused on transcriptional changes of key targets and coordinately regulated gene networks after mKO and loss of REV-ERBα [7,17]. REACTOME pathway enrichment analysis [20] performed on 931 differentially expressed genes in muscles from mKO mice [7] uncovered changes in canonical clock-related pathways and general perturbations in fatty acid, triglyceride (TG), and phospholipid metabolism (Fig 2A).

Accordingly, the top-ranking pathways affected by loss of *Bmal1* included genes regulated by “BMAL1:CLOCK” and “Circadian Clock.” Moreover, we noted that most muscle clock-dependent circadian genes [17] displayed transcriptional changes one might expect from direct muscle BMAL1 targets and suggested very specific yet disparate metabolic consequences. For example, *Slc7a2* and *Coq10b* were both highly oscillatory genes in wild-type (WT) muscles, and both completely lost 24-hr oscillation in mKO muscles (Fig 2B). *Slc7a2* codes for a cationic amino acid transporter, whereas *Coq10b* is thought to code for a scaffold/chaperone protein regulating coenzyme Q localization within the inner mitochondrial membrane [21]. According to our cistrome data, both genes may be direct functional targets of BMAL1, and their altered expression profiles in mKO muscles suggest that impaired cationic amino acid transport [22] and coenzyme Q deficiency might be consequences of muscle clock perturbation/misalignment.

Likewise, REV-ERBα and REV-ERBβ displayed drastically disrupted diurnal expression [7] and transcriptional activity in mKO muscles (Fig 2A). While *Rev-erbα* and *Rev-erbβ* are among the highest expressed nuclear receptors in fast glycolytic and slow oxidative muscles (S3A Fig), REV-ERBα and REV-ERBβ protein levels are restricted to an 8-hr temporal window at the end of the light phase, around ZT8–ZT12 (Fig 2C). Loss of REV-ERBα/β in mKO muscles was especially apparent at the protein level, with normal accumulation of REV-ERBα and REV-ERBβ completely abolished in mutant muscles. This underscores the fact that muscle-specific *Bmal1* knockout (KO) mice are also, in essence, muscle-specific *Rev-erbα* and *Rev-erbβ* double-KO mice, in agreement with enrichment analysis (Fig 2A). Accordingly, we noted derepression (i.e., increased expression) of canonical REV-ERB target genes (Fig 2D)—like *p21* (*Cdkn1a*) [23] and *Clock* [7,24]—and previously identified skeletal muscle HDAC3 targets [25] including *Ppp1r3c*, a master regulator of glycogen synthesis; *Mybph*, coding for a protein thought to be involved in autophagosome maturation [26]; *Ampd1*, the predominant skeletal muscle adenosine monophosphate (AMP) deaminase; and *Thra* coding for THRα.

REV-ERBα/β are known to regulate lipid metabolism in peripheral tissues [12,15,27], including skeletal muscle [28,29], but a comprehensive understanding of their direct muscle targets, their cross-talk with other nuclear hormone receptors, and the effects of muscle-specific loss of function remain largely unknown. REV-ERBα is known to directly compete with and inhibit TR/RXR binding at target sites during muscle differentiation [30], and REV-ERBα and PPAR signaling pathways are likewise known to converge [31]. PPARs are nuclear hormone receptors that mediate adaptive metabolic responses, including increased lipid oxidation and amino acid catabolism in muscle [32] following their activation by endogenous or dietary lipids or lipid derivatives [33]. REV-ERBα/β is known to repress transactivation of some PPAR targets by competitively binding to nearby genomic sites in a concentration-dependent manner [34].

Consistent with motif enrichment of BMAL1 and REV-ERBα peaks (Fig 1D and S2E Fig), PPAR signaling was also among the top-ranking pathways impacted by mKO (Fig 2A). Furthermore, mKO muscles showed an altered gene expression profile reminiscent of acute pharmacological activation using the PPARδ receptor agonist GW501516 [35]. In particular, we noticed significantly increased expression of major regulatory genes involved in mobilizing intracellular lipid stores (*Atgl*/*Pnpla2*) and channeling fatty acids from lipid droplets to the mitochondria for oxidation (*Plin5*) (Fig 2E). Additionally, mKO muscles showed significantly increased expression of genes involved in fatty acid transport (*Fatp-1/Slc27a1*), activation of fatty acids to corresponding acyl-CoAs (*Acsl1*), and finally breakdown (*Acadm*) and oxidation (*Hadha*) of fatty acids. However, we observed no major changes in diurnal expression of PPARs in fast or slow muscles (S3B Fig), suggesting that increased PPAR target expression in mKO muscles results from increased presence of endogenous ligands and/or PPAR activation. Highlighting potential sites for cross-talk/competition between REV-ERBα and PPARs, we noted REV-ERBα peaks near known PPAR regulatory elements (PPREs) in promoters of *Plin5* and *Acsl1* [36,37].

To gain more mechanistic insight into how BMAL1 and REV-ERBα regulate muscle targets, and their functional roles, we further stratified our cistrome data according to direction of expression changes in mKO muscles (Fig 2G and S1 Table). Hundreds of BMAL1 and REV-ERBα targets showed differential regulation, suggesting a direct link between loss of BMAL1 and REV-ERBα and gene expression changes in mKO muscles. According to pathway enrichment analysis [20], down-regulated BMAL1 and REV-ERBα targets reflected common regulation of circadian clock genes and transforming growth factor beta (TGFβ) signaling components. Down-regulated BMAL1 targets were further associated with mitogen-activated protein kinase (MAPK) and other stress response pathways, whereas down-regulated REV-ERBα targets were associated with nuclear factor kappa B (NFκB) activation. Interestingly, up-regulated target genes showed less cooperation between BMAL1 and REV-ERBα and suggested more specific regulation of calcium and Wnt signaling by BMAL1, while REV-ERBα showed specificity for the GR signaling pathway and heme biosynthesis (Fig 2G). We further corroborated REV-ERBα-specific association with GR targets by visually inspecting prominent REV-ERBα peaks with high enrichment scores near selected GR-regulated genes (S2F Fig), including transcriptional regulators like *Fos* and *Trp53* [38].

When glucose availability is low, skeletal muscle adapts to preferentially increase the uptake and oxidation of lipids [39] while coordinately increasing protein degradation for production and release of amino acids destined for gluconeogenesis [40]. This transcriptionally regulated proteolytic process is under tight hormonal control [41] and is largely mediated by synergism between ligand-bound GR and activated members of the forkhead box O (FOXO) family of transcription factors [42]. REV-ERBα is also speculated to cross-talk with GR in adult muscle [43]. Accordingly, several putative REV-ERBα/GR target genes uncovered by our muscle cistrome showed transiently increased expression in mutant muscles, particularly during the hours around the light–dark transition (Fig 2F, S2F and S3C Figs), coinciding with peak levels of endogenous glucocorticoids [44] and loss of REV-ERBα protein in mKO muscle (Fig 2C). These included major mediators of muscle protein turnover, like *MuRF-1* (*Trim63*) and *Atrogin-1/MAFbx* (*Fbxo32*)—two E3 ubiquitin ligases involved in targeting myofibrillar proteins for degradation—and polyubiquitin-C (*Ubc*), a major determinant of the intracellular ubiquitin pool. In addition, we found increased expression of several genes coding for proteasome subunits (*Psmb3*, *Psmb6*, *Psmc4*, *Psmd14*), as well as master regulators of autophagy (*Trp53*, *Atg12*, and *Ulk1*), and finally *Snat2* (*Slc38a2*), the highly energized System A amino acid transporter increased by cortisol [45] and amino acid starvation [46].

Collectively, our data suggest that one role of the muscle clock is to anticipate the feeding phase by direct REV-ERB-mediated repression of important targets involved in the mobilization and metabolism of lipids and amino acids. Furthermore, muscle-specific loss of BMAL1, and thus REV-ERBα, is associated with increased expression of direct REV-ERBα targets and coordinated gene networks known to regulate lipid and amino acid metabolism, muscle protein turnover, and autophagy.

### Muscle-specific loss of BMAL1 alters 24-hr lipid and amino acid metabolism

**Fig 3:**
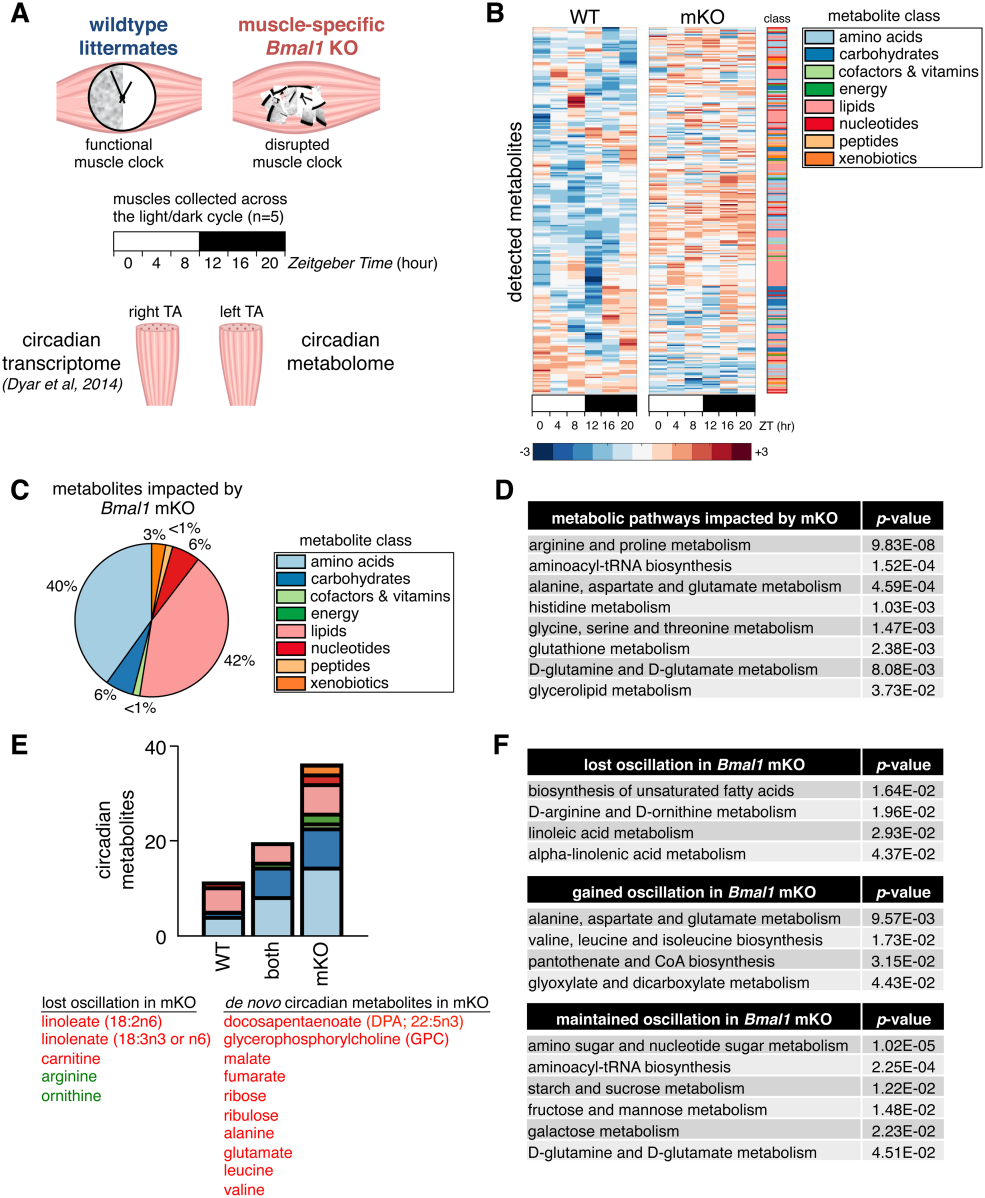
Global metabolite alterations associated with muscle-specific clock disruption. (A) Experimental design showing integration of 24-hr metabolomics data with transcriptomics data from contralateral muscles. (B) Global 24-hr metabolomics of TA muscles from WT and mKO mice. Heatmap shows mean scaled abundance (*n* = 5 × timepoint × group) of detected metabolites across the light/dark cycle (white/black bar). Metabolites are sorted by phase according to WT muscle and aligned between groups to show effect in mKO. (C) Class distribution of metabolites significantly impacted by muscle-specific *Bmal1* KO (genotype effect *p* < 0.05, mixed effects model). (D) Integrated pathway enrichment analysis combining metabolomics and transcriptomics data. (E) Class distribution of metabolites oscillating with a 24-hr period (*p* < 0.05, JTK_CYCLE; red = significantly increased in mKO muscles; green = significantly reduced). (F) Integrated pathway enrichment and topology analysis of 24-hr cycling metabolites. Underlying data can be found in supporting file S1 Data. KO, knockout; mKO, myocyte-specific loss of BMAL1; TA, tibialis anterior; WT, wild type; ZT, Zeitgeber hour.

To understand how local diurnal rhythms of muscle metabolism are impacted by differential expression of gene programs described above, we performed global 24-hr metabolite profiling of tibialis anterior (TA) muscles from mKO mice and their WT littermates (Fig 3A). Tissues were collected every 4 hr across the light/dark cycle, and metabolites were profiled by mass spectrometry (liquid chromatography/mass spectrometry [LC/MS] and gas chromatography/MS [GC/MS], see Materials and methods). Importantly, we used the contralateral muscles from the same cohort of mice used for 24-hr transcriptomics [7], allowing us to directly correlate diurnal muscle metabolite alterations with diurnal changes in muscle gene expression.

A large proportion of metabolites showed clear alterations in diurnal oscillation and abundance in mKO muscles (Fig 3B and S3 Table). Lipids and amino acids showed the greatest impact from clock disruption (Fig 3C), comprising 42% and 40%, respectively, of all significantly altered metabolites (genotype effect *p* < 0.05, mixed effects model). Integrated pathway enrichment analysis [47] likewise revealed substantial alterations in amino acid and lipid metabolism pathways (Fig 3D). In particular, we noted that several anabolic pathways directly linked to glycolysis were affected, including alanine, glycine, serine, and glycerolipid metabolism.

Identification of 24-hr period oscillating metabolites (JTK_Cycle *p* < 0.05) revealed a completely unexpected >2-fold increase in circadian metabolites in mKO muscles (Fig 3E). De novo oscillating metabolites identified by this analysis were predominantly amino acids—including alanine, glutamate, leucine, and valine—but also included pentoses (ribose and ribulose), tricarboxylic acid (TCA) cycle intermediates (malate and fumarate), and essential lipids (docosapentaenoate 22:5n3). Relatively few metabolites lost 24-hr oscillation after clock disruption, yet these were again mainly lipid-related metabolites like carnitine, essential lipids (linoleate 18:2n6 and linolenate 18:3n3 or 6), and amino acids (arginine and ornithine). Our integrated pathway enrichment and topology analysis [47] of these 24-hr oscillating metabolites reflected a gain of oscillation in pathways related to alanine, glutamate, and branched-chain amino acid (BCAA) metabolism; pantothenate and CoA biosynthesis; and glyoxylate and dicarboxylate metabolism (Fig 3F). Conversely, oscillation of pathways involved in the biosynthesis of unsaturated fatty acids, arginine and ornithine metabolism, and linoleic and linolenic acid metabolism all showed impaired 24-hr oscillation after loss of BMAL1. Finally, pathways involved in carbohydrate metabolism (starch and sucrose, fructose and mannose, and galactose metabolism), amino and nucleotide sugar metabolism, and aminoacyl-tRNA biosynthesis all retained 24-hr oscillation in mKO muscles.

In summary, our integrated metabolic analyses identified lipids and amino acids as the metabolite classes showing the greatest impact from loss of BMAL1, with generally increased abundance and increased 24-hr oscillation patterns in mKO muscles. In extension of previously reported impairments to glucose metabolism [7,48,49], our data demonstrate an important role for BMAL1 in the regulation of lipid and amino acid metabolism.

### Impaired neutral lipid storage and accumulation of bioactive lipids in mKO muscles

Skeletal muscle TGs are an important and readily available local fuel reserve [50], especially after long periods of activity, when muscle glycogen stores are depleted [51]. In humans, replenishment of muscle TG stores can occur within hours [51,52], and content is increased when carbohydrate availability is low and circulating lipids are elevated, like during fasting or high-fat diet [53]. To gain a general perspective on diurnal rhythms of muscle neutral lipid storage, we profiled TGs at 2 physiologically relevant time points in WT and mKO muscles.

**Fig 4:**
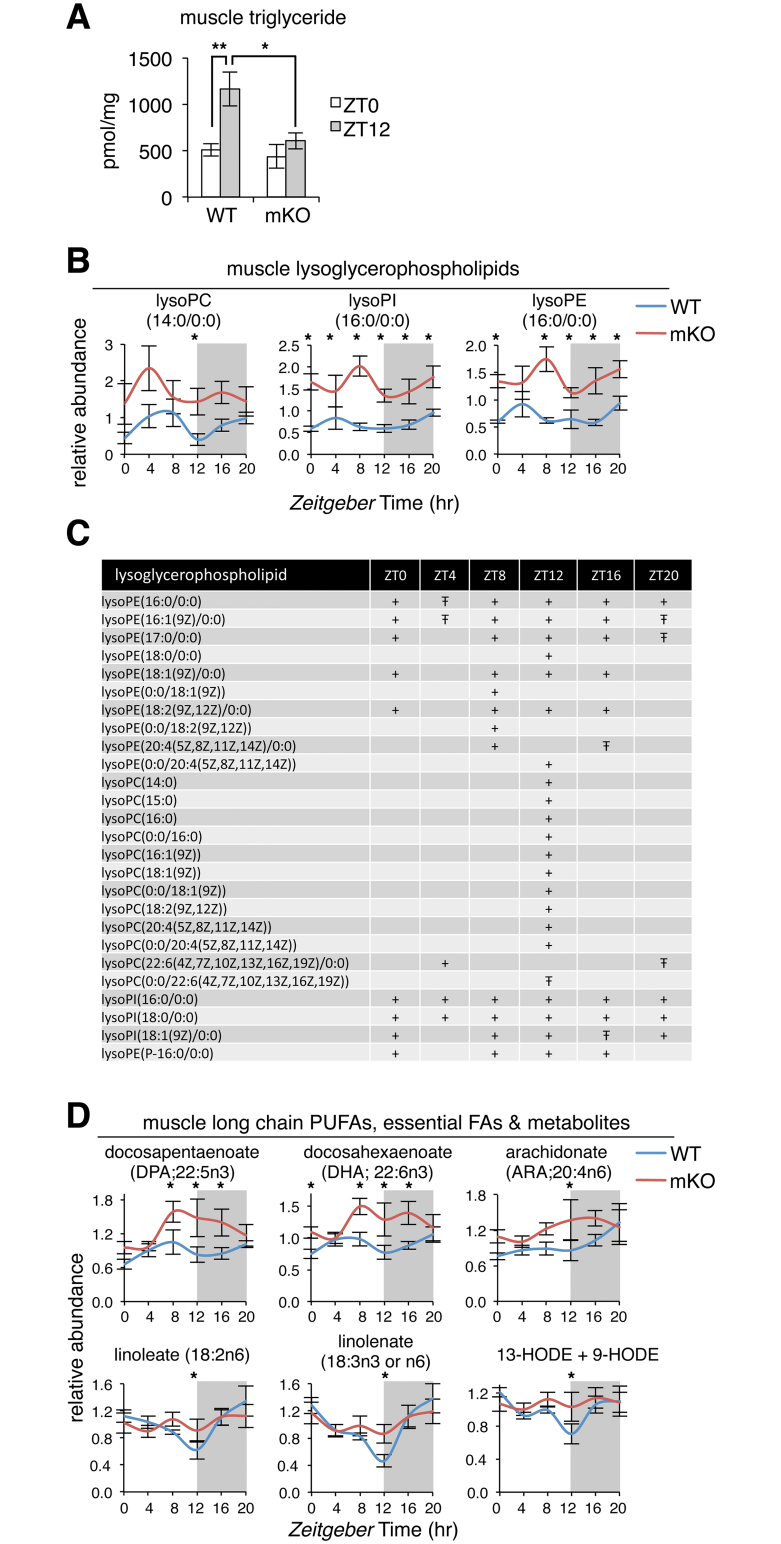
Impaired storage of neutral lipids and accumulation of bioactive lipids in mKO muscles. (A) Muscle triglyceride extracted from gastrocnemius muscles, quantified by HPLC, and normalized to cell protein (mean ± SEM; *n* = 5 × group × timepoint; **p < 0.01, *p < 0.05, Student’s *t* test). (B) Diurnal variations of selected lysoglycerophospholipids in WT and mKO TA muscles (mean ± SEM; *n* = 5 × group × timepoint; *p < 0.05, repeated measures ANOVA). (C) Temporal distribution of significantly increased lysoglycerophospholipids comparing WT and mKO TA (+*p* < 0.05, Ŧ*p* < 0.1, repeated measures ANOVA). (D) Diurnal variations of selected long-chain PUFAs, essential FAs, and related metabolites in WT and mKO TA muscles (mean ± SEM; *n* = 5 × group × timepoint; *p < 0.05, repeated measures ANOVA). Underlying data can be found in supporting files S1 Data and S3 Table. FA, fatty acid; HPLC, high-performance liquid chromatography; HODE, hydroxyoctadecadienoate; lysoPC, lysoglycerophosphocholine; lysoPE, lysoglycerophosphoethanolamine; lysoPI, lysoglycerophosphoinositol; mKO, myocyte-specific loss of BMAL1; PUFA, polyunsaturated fatty acid; TA, tibialis anterior; WT, wild type; ZT, Zeitgeber time.

Quantitative lipidomics performed at “lights on” (ZT0) and “lights off” (ZT12, 12 hr later) revealed a 2-fold accumulation of total TG in WT muscles at ZT12 (Fig 4A), the end of the physiological fasting phase. This accumulation was completely abolished in mKO muscles, with total TG content remaining static at basal levels. We noticed that mKO mice showed a normal daily rhythm of food intake, with completely normal distribution of feeding time and amount (S4A Fig). Similarly, plasma non-esterified fatty acids (NEFAs), lactate, and ketone bodies (β-hydroxybutyrate [β-OH-B] and acetoacetate [AcAc]) were all comparable to WT levels in sedentary mice independently of whether they had been fasted (4 hr, collected at ZT11) or fed (collected at ZT14), as well as in endurance-trained mice after 1 hr of treadmill running (S4B Fig). Reduced TG accumulation in mKO muscles therefore seems to reflect local alterations in muscle lipid metabolism rather than differences in feeding behavior or interorgan lipid fluxes.

Profiling individual TG species revealed a general reduction of TG accumulation in mKO muscles at ZT12 rather than specific changes in esterified fatty acid composition. All TG species were increased around 2-fold at ZT12 compared to ZT0 in WT muscles, and all were drastically reduced at ZT12 in mKO muscles (S4C Fig). At the same time, the total distribution of TG species was completely normal in mKO muscles (S4D Fig), with over 92% of all TG species containing 48, 50, 52, or 54 carbons and multiple unsaturated fatty acids, regardless of time point or genotype.

While neutral lipid storage was impaired in mKO muscles, we noted a striking general increase of various bioactive lipids involved in signaling and inflammation. These included several lysoglycerophospholipids (lysoPLs) comprising a wide spectrum in terms of hydrophilic and hydrophobic moieties (Fig 4B and 4C; S3 Table). While many lysoPLs were significantly increased in mKO muscles, ZT12 emerged as a particularly important time point, with 85% significantly increased in mKO muscles during the light–dark transition, including the lysophosphatidylcholine LPC (16:0), thought to be an endogenous PPAR ligand [54].

Circadian metabolomics also revealed a transient increase in mKO muscles of many long-chain polyunsaturated fatty acids (PUFAs; docosapentaenoate 22:5n3 and docosahexaenoate 22:6n3), essential fatty acids (linoleate 18:2n6 and linolenate 18:3n3 or n6), and oxidation- and inflammation-associated metabolites (13-hydroxyoctadecadienoate + 9- hydroxyoctadecadienoate, dihomo-linolenate 20:3n3 or n6, and arachidonate 20:4n6) (Fig 4D; S3 Table). Importantly, most of these lipid species are also thought to be endogenous PPAR ligands [55] and so may contribute to increased expression of PPAR targets in mKO muscle. Taken together, mKO muscles showed reduced neutral lipid storage (TG) and a significant accumulation of bioactive lipids, particularly during the hours around the light–dark transition.

### Increased glucogenic amino acids in mKO muscles linked to TCA cycle anaplerosis

**Fig 5:**
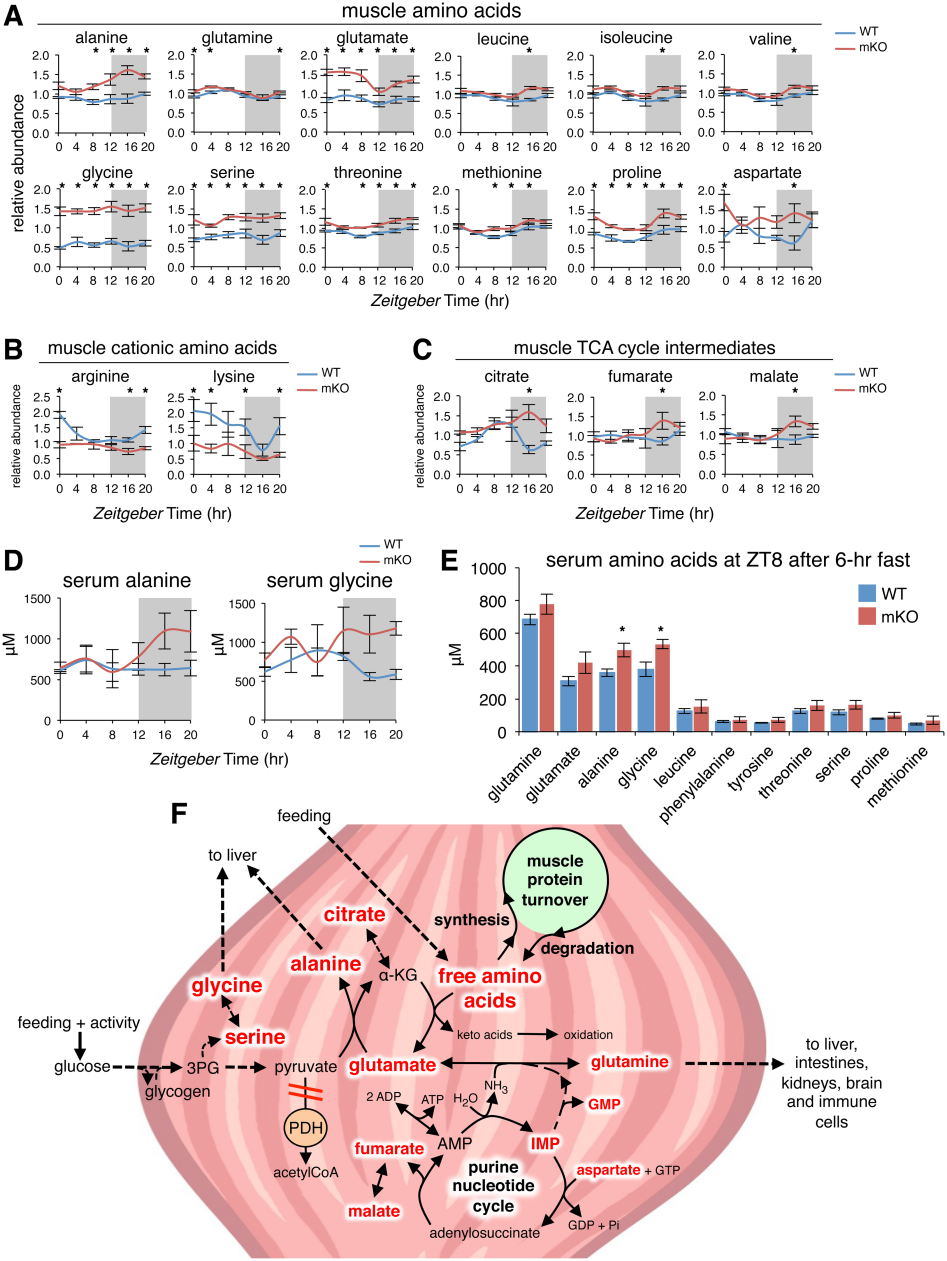
Increased amino acids in mKO muscles linked to TCA cycle anaplerosis. (A-C) Diurnal levels of selected amino acids (A), cationic amino acids (B), and TCA cycle intermediates (C) in WT and mKO TA muscles (mean ± SEM; *n* = 5 × group × time point; *p < 0.05, repeated measures ANOVA). (D) Serum alanine (genotype effect *p* < 0.05) and glycine (genotype effect *p* < 0.01) from ad libitum fed mice (mean ± SEM; *n* = 3–4; 2-way ANOVA with Bonferroni correction). (E) Serum amino acids at ZT8 after a 6-hr fast. (mean ± SEM; *n* = 3–4; *p < 0.05 Student’s *t* test). (F) Simplified scheme showing interrelationships between increased amino acids, purine nucleotides, and TCA cycle intermediates in mKO muscle (indicated by red text). Increased glycolytic flux in the context of impaired PDH activity [7] may divert glycolytic intermediates to alternative biosynthetic pathways, including serine, glycine, and alanine formation. Circadian metabolomics data also suggest mKO muscles undergo increased rates of protein turnover, increased formation and release of alanine and glycine, and increased anaplerotic flow of carbon into an expanded TCA cycle via citrate, fumarate, and malate. Underlying data can be found in supporting file S1 Data. 3PG, 3-phosphoglycerate; α-KG, alpha-ketoglutarate; AMP, adenosine monophosphate; GDP, guanosine diphosphate; GTP, guanosine triphosphate; mKO, myocyte-specific loss of BMAL1; PDH, pyruvate dehydrogenase; TA, tibialis anterior; TCA, tricarboxylic acid; WT, wild type; ZT, Zeitgeber time.

Amino acids were the other major metabolite class significantly impacted by muscle-specific loss of BMAL1. In particular, we noted a significant increase of glucogenic amino acids in mKO muscles (Fig 5A and S5A Fig). Alanine, glutamine, glutamate, glycine, serine, threonine, methionine, proline, and aspartate were all significantly increased between 20% and 300% at multiple time points throughout the light/dark cycle in mKO muscles. At ZT16, the BCAAs leucine, isoleucine, and valine, in addition to cysteine, were all significantly increased 25%–35% in mKO muscles relative to WT. In contrast, the cationic amino acids arginine and lysine were significantly reduced 30%–60%, with 24-hr oscillation severely blunted in mKO muscles (Fig 5B), concordant with reduced *Slc7a2* at the end of the dark phase (Fig 2B). Finally, aromatic amino acids phenylalanine, tyrosine, and tryptophan remained unchanged (S5A Fig). We also noted increased levels of several metabolites linked to intermediary amino acid metabolism, including 5’-inosine monophosphate (5’-IMP) and 5’-guanosine monophosphate (5’-GMP) (S5B Fig), both regulators of the purine nucleotide cycle [56], as well as the TCA cycle intermediates citrate, fumarate, and malate (Fig 5C).

To investigate whether the increase in free amino acid levels observed in mKO muscles are directly correlated with differences in circulating amino acids, we quantified serum amino acids in ad libitum–fed WT and mKO mice across the light/dark cycle. Importantly, mKO mice showed persistently increased serum alanine and glycine levels throughout the dark phase (Fig 5D), despite normal feeding behavior and blood lipid profiles (S4A Fig). These reflect the largest differences we observed in mKO muscles (Fig 5A) and are in agreement with transiently increased expression of REV-ERBα targets (Fig 2F) linked to the temporally restricted production and release of these major [41] glucogenic precursors. To validate these findings under a more controlled nutritional state, we also examined serum amino acids after a 6-hr fast at ZT8, during the normal physiological fasting+rest phase when diurnal insulin levels are lowest [57]. While serum amino acids were generally increased in mKO mice, only alanine and glycine reached significance (Fig 5E).

Contextualizing our results from a temporal perspective, we conclude that increased amino acids, purine nucleotides, and TCA cycle intermediates in mKO muscles are all closely related (Fig 5F). We noticed diurnal alanine levels increased substantially in mKO muscles as glutamate levels decreased, starting around ZT8–ZT12 and coinciding with inhibition of pyruvate dehydrogenase (PDH) in mKO muscles [7]. Increased alanine in mKO muscles thus likely reflects increased mass-action conversion of pyruvate and glutamate into alanine and α-ketoglutarate, a freely reversible and near-equilibrium reaction catalyzed by alanine aminotransferase [58]. Quantitatively, this is the most important anaplerotic reaction contributing to expansion of the TCA intermediate pool at the start of exercise [59]. In support of this interpretation, peak alanine levels in mKO muscle increased 83% compared to control levels at ZT16 and coincided with peak TCA intermediates citrate, fumarate, and malate, which increased respectively 251%, 62%, and 52% at ZT16.

Alanine is a particularly important precursor for hepatic gluconeogenesis [41], and its production and export reflect nutritional state more closely than that of glutamine, the other major amino acid produced and released by skeletal muscle [60]. Alanine increases the most during the earliest phases of starvation [61] and correlates with increased muscle protein degradation [41,58,62]. The carbon required for alanine formation is derived mostly from circulating glucose and muscle glycogen, while the nitrogen comes from the catabolism of other amino acids, mainly BCAA, as well as others [63,64]. The particularly large increase of alanine in mKO muscles thus indicates an energy deficit and related increase in proteolysis in mKO muscles during this time [65]. However, feeding is normal in mKO mice (S4A Fig), and muscles from mKO mice show normal or even slightly increased content of muscle glycolytic intermediates, AKT phosphorylation, and glycemia [7]. Therefore, fasting, or some other systemic starvation signal (i.e., alterations in circulating glucose or insulin), cannot explain increased alanine and other amino acids in mKO muscles. Instead, their accumulation must reflect a local defect in energy sensing or metabolism related to loss of BMAL1.

### Increased lipid and amino acid oxidation in mKO muscles linked to oxidative stress, altered mitochondrial function, and metabolic inefficiency

**Fig 6:**
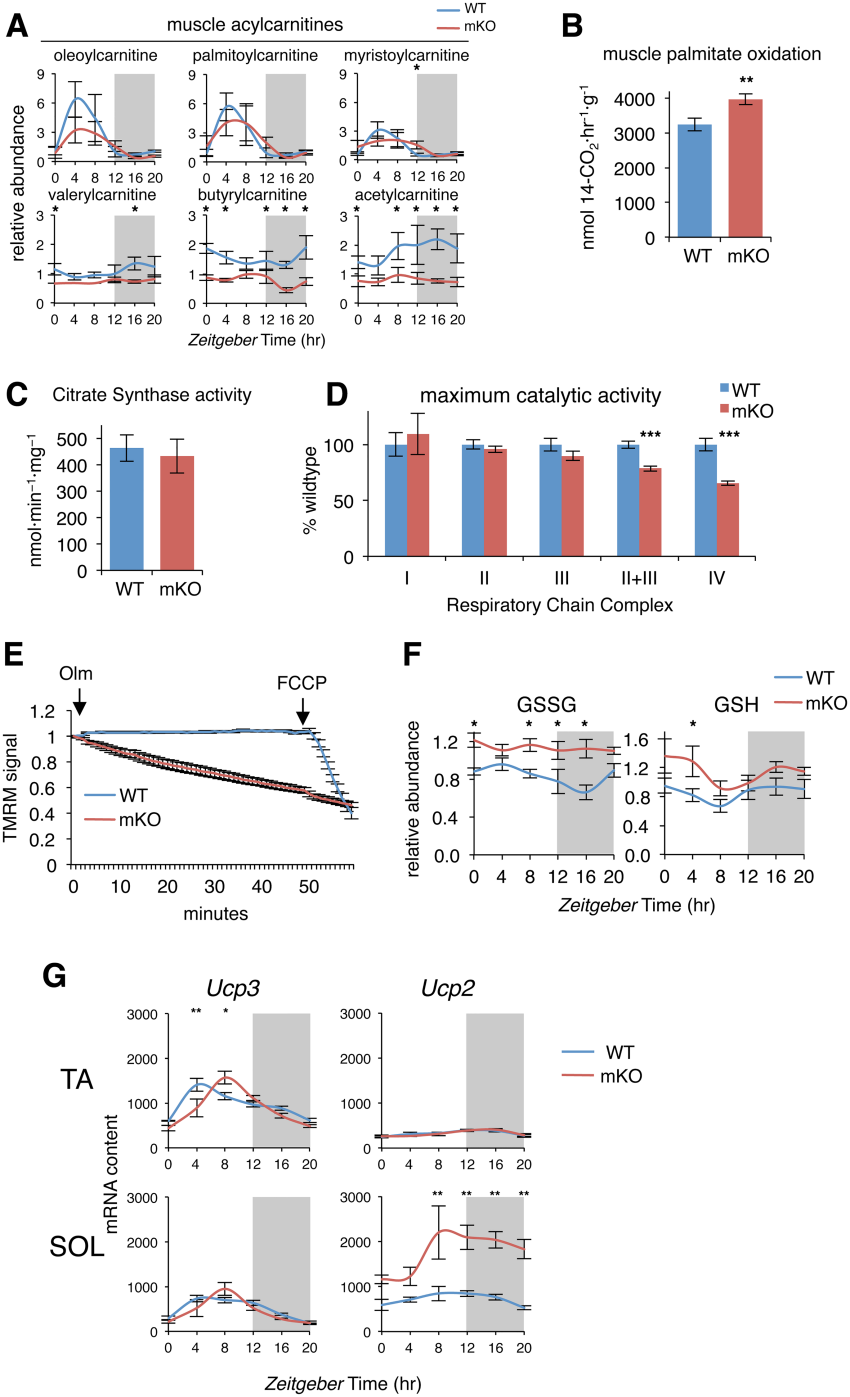
Increased lipid oxidative capacity yet reduced mitochondrial efficiency in mKO muscles. (A) Diurnal levels of selected acylcarnitines in TA muscles (mean ± SEM; *n* = 5 × group × time point; *p < 0.05, repeated measures ANOVA). (B) Palmitate oxidation in gastrocnemius homogenates (mean ± SEM; *n* = 12–13; **p < 0.01, Student’s *t* test). (C) CS activity in vastus lateralis muscle extracts (mean ± SEM; *n* = 18). (D) Maximum catalytic activity of Respiratory Chain Complex I (NADH:ubiquinone oxidoreductase; *n* = 6), Complex II (succinate dehydrogenase; *n* = 18), Complex III (decylubiquinol cytochrome *c* oxidoreductase; *n* = 18), Complex II+III (succinate cytochrome *c* reductase; *n* = 18), and Complex IV (cytochrome *c* oxidase; *n* = 18) in vastus lateralis muscle extracts (mean% relative to WT ± SEM; ***p < 0.001, Student’s *t* test). (E) Real-time fluorescence of isolated flexor digitorum brevis muscle fibers loaded with TMRM and imaged every 60 s; data expressed relative to initial fluorescence; 5 μM Olm and 4 μM FCCP were added where indicated (mean ± SEM; *n* = 21–24 fibers isolated from 5 animals per group). (F) Diurnal levels of GSSG and GSH in mKO and WT TA muscles (mean ± SEM; *n* = 5 × group × time point; *p < 0.05, repeated measures ANOVA). (G) Diurnal expression in TA and SOL muscles determined by microarray and plotted as absolute expression levels (*n* = 3 × time point; mean ± SEM *p = 0.05, **p = 0.01, 2-way ANOVA with Bonferroni correction). Underlying data can be found in supporting files S1 Data and at Gene Expression Omnibus (accession number GSE43071). CS, citrate synthase; FCCP, carbonylcyanide-p-trifluoromethoxyphenyl hydrazone; GSH, reduced glutathione; GSSG, oxidized glutathione; mKO, myocyte-specific loss of BMAL1; Olm, oligomycin; SOL, soleus; TA, tibialis anterior; TMRM, tetramethylrhodamine methyl ester; WT, wild type.

Accumulation of bioactive lipids and amino acids can be symptomatic of mitochondrial dysfunction or may simply reflect an imbalance between oxidative substrate supply and energy demand [66,67]. We uncovered several complementary lines of evidence that suggest mKO muscles have increased lipid and amino acid oxidative capacity and altered mitochondrial function. According to our 24-hr metabolome, long-chain acylcarnitines showed distinct diurnal fluctuations in WT muscles, with a peak during the rest+fasting phase and reduced levels during the activity+feeding phase (Fig 6A). These diurnal oscillations were similar in mKO muscles, except for slightly reduced peak oscillation for some species, like oleoylcarnitine and palmitoylcarnitine, and a modest increase at ZT12, which was statistically significant only for myristoylcarnitine.

In contrast, all medium- and short-chain acylcarnitines were significantly reduced at various timepoints. This was particularly striking for acetylcarnitine (Fig 6A) but also branched-chain acylcarnitines derived from BCAA catabolism, including isobutyrylcarnitine, 2-methylbutyroylcarnitine, isovalerylcarnitine, propionylcarnitine, and succinylcarnitine (S3 Table). Reduced levels of short- and medium-chain acylcarnitines relative to essentially normal long-chain acylcarnitines and increased BCAA (only at ZT16) may reflect increased lipid and BCAA catabolism, with faster transit of carbon chains through the pathways. Increased lipid and amino acid catabolism could also supply increased acetyl CoA or succinyl CoA, in agreement with TCA cycle anaplerosis (Fig 5C). To measure lipid catabolism rates, we quantified [1-^14^C]palmitate oxidation to [1-^14^C]CO_2_ in whole gastrocnemius muscle homogenates collected at ZT12. Compared to control muscles, mKO muscles showed a clear and significant 22% increase in palmitate oxidation rate (Fig 6B), in agreement with transcriptional changes of lipid metabolism genes (Fig 2F), and suggesting increased β-oxidation capacity.

Increased oxidative capacity in mKO muscles can result from a combination of factors, including increased mitochondrial content, increased activity of respiratory chain complexes, or increased proton conductance related to mitochondrial membrane potential (Δ*ψ*_m_) [68,69]. To quantify mitochondrial content in control and mKO muscles, we measured citrate synthase (CS) activity in muscle homogenates and found no differences (Fig 6C). We next assayed enzymatic activities of respiratory chain complexes I–IV and found maximum catalytic activity of complexes I, II, and III were likewise unchanged in mKO muscles (Fig 6D). However, we detected a highly significant 20% reduction in complex II+III activity in mKO muscles, suggesting a mild coenzyme Q deficiency, consistent with reduced *Coq10b* (Fig 2B), whereas complex IV activity was significantly reduced around 30%.

To investigate alterations in Δ*ψ*_m_, we performed real-time fluorescence imaging of Δ*ψ*_m_ in isolated muscle fibers loaded with the potentiometric fluorescent dye tetramethylrhodamine methyl ester in the presence of oligomycin A, an inhibitor of the mitochondrial F_1_F_O_-ATP synthase. In this system, maintenance of Δ*ψ*_m_ relies on the reverse activity of ATP synthase. Oligomycin addition caused no change in Δ*ψ*_m_ in control muscle fibers (Fig 6E), whereas marked depolarization was induced by the protonophore carbonylcyanide-p-trifluoromethoxyphenyl hydrazone (FCCP). While WT mitochondria were polarized and metabolically efficient, progressive mitochondrial depolarization was apparent in mKO myofibers. Addition of FCCP had little impact on these already depolarized cells, suggesting mKO muscle mitochondria have reduced Δ*ψ*_m_ and a general loss of coupling efficiency.

Mild uncoupling of skeletal muscle mitochondria is thought to mitigate potentially damaging oxidative stress [68]. Examining various markers of oxidative stress, we noted that oxidized glutathione (GSSG) oscillated around 30% over 24 hr in control muscles. GSSG was normally highest during the rest+fasting phase and remained lowest during the activity+feeding phase (Fig 6F). However, in mKO muscles, GSSG remained constitutively increased across the light/dark cycle, suggesting increased buffering demand. On the other hand, reduced glutathione was only slightly increased in mKO muscles, and the difference was significant only at ZT4. Another biomarker of oxidative stress resulting from peroxidation of PUFAs, 4-hydroxy-2-nonenal, also showed only a slight trend for increased abundance during the fasting+rest phase and otherwise remained within a normal diurnal physiological range (S6A Fig). Overall, while chronic loss of BMAL1 appears to be associated with a mild and transient increase in oxidative stress, this appears to be sufficiently buffered through a combination of mild uncoupling and various endogenous antioxidant systems.

Mild oxidative stress is known to increase protein degradation in muscle cells by increasing expression and activity of the ubiquitin-proteasome system [70] and so may also be causally linked to increased amino acids (Fig 5A). Mild uncoupling was previously shown to increase 2-fold by starvation in rat skeletal muscle mitochondria and is mediated by uncoupling proteins in the presence of fatty acids and in response to coenzyme Q–generated superoxide [71]. We noted *Ucp3* expression was significantly increased at ZT8 in mKO TA muscles compared to WT, while *Ucp2* expression was massively induced at all time points in mKO soleus, peaking around ZT8 (Fig 6G).

Our results indicate that reduced energy efficiency and increased supply of oxidative substrates are linked to a mild increase in oxidative stress and a transcriptionally regulated mild uncoupling in mKO muscles. However, evidence suggests these changes should be considered in terms of altered function rather than dysfunction. Increased oxidative capacity with increased uncoupling from ATP production is observed in muscles of endurance-trained athletes [72,73] and in rodents fed a high-fat diet [74], with enhanced sensitivity of mitochondrial uncoupling to fatty acids seen in both cases. As such, one might interpret reduced coupling efficiency of mKO muscle mitochondria similarly as a consequence of increased lipid metabolism. In any case, we saw no differences in endurance capacity after a graded exercise tolerance test to exhaustion (S6B Fig). Using glycemia <76 mg/dl to establish fatigue [75], mKO mice showed neither deficit nor advantage in terms of exercise endurance capacity, running more than 2 km in around 2 hr, just as their WT littermates. Normal exercise endurance in mKO mice likely reflects normal 24-hr glycogen stores in muscle and liver (S6C Fig), since liver glycogen stores are known to be the main determinant of exercise endurance capacity in mice [76,77].

### Muscle-specific loss of BMAL1 causes fat-to-lean body mass partitioning, increased rates of muscle protein synthesis, and increased EE

**Fig 7:**
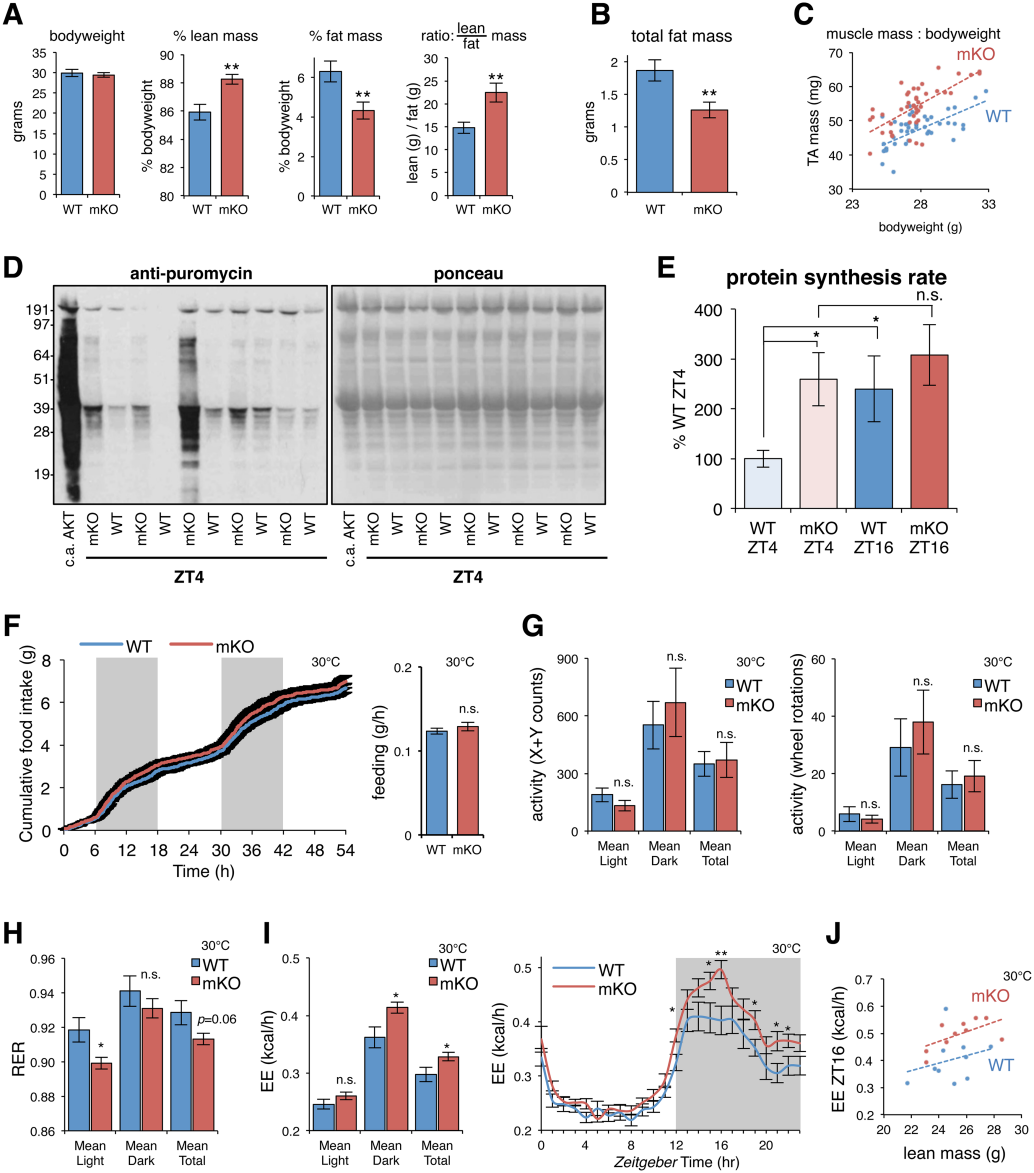
Muscle-specific loss of BMAL1 associated with fat-to-lean mass partitioning, increased rates of muscle protein synthesis, and increased EE. (A-B) *n* = 11–12 mice; 5-mo-old male littermates; mean ± SEM; **p < 0.01, Student’s *t* test. (A) Normal bodyweight, increased percent lean mass, reduced percent fat mass, and increased lean-to-fat mass ratio in mKO mice. (B) About 30% reduced total body fat. (C) Increased muscle mass relative to bodyweight (TA; *n* = 44 five-mo-old male littermates; dashed line indicates mean trend line). (D-E) In vivo protein synthesis rates of TA measured by IV-SUnSET. (D) Representative image of western blot analysis for puromycin-labeled peptides followed by ponceau staining to verify equal protein loading. TA from a transgenic mouse with c.a.AKT was used as positive control. (E) Quantification of puromycin-labeled peptides expressed as a percentage of the values obtained in WT ZT4 (mean ± SEM; *n* = 10, ZT4; *n* = 5, ZT16; *p < 0.05, Student’s *t* test). (F-I) Effects of muscle-specific *Bmal1* mKO on (F) food intake, (G) locomotor activity (X + Y axis counts and running wheel rotations), (H) RER (VCO2/VO2), and (I) EE all measured at thermoneutrality (30 °C). *n* = 10 four-mo-old male littermates; mean ± SEM; *p < 0.05, Student’s *t* test; ANCOVA genotype effect *p* = 0.046 (total EE) and *p* = 0.023 (dark phase EE) when considering body weight, lean mass, and fat mass as covariates. (J) Relationship between EE and lean mass at ZT16 (*n* = 10; dashed line indicates mean trend line). Underlying data can be found in supporting file S1 Data. BMAL1, brain and muscle ARNT-like protein 1; c.a.AKT, constitutively active AKT; EE, energy expenditure; IV-SUnSET, in vivo surface sensing of translation; mKO, myocyte-specific loss of BMAL1; n.s., not significant; RER, respiratory exchange ratio; TA, tibialis anterior; VCO2, volume carbon dioxide produced; VO2, volume oxygen consumed; WT, wild type; ZT, Zeitgeber time.

We previously showed that mKO mice have increased muscle mass yet normal bodyweight compared to their WT littermates [7]. Closer examination confirmed [48] significant differences in body composition in mKO mice (both fat and lean tissue mass). While bodyweight of adult mice was normal, mKO mice showed increased lean mass and reduced fat mass according to EchoMRI, ultimately translating into an increased lean-to-fat body mass ratio (Fig 7A). We found this was due to a combination of about 30% reduction in total body fat mass (Fig 7B) and a 10%–20% increase in muscle mass (Fig 7C).

Reduced peripheral fat mass in mKO mice is in agreement with increased lipid oxidation and reduced lipid storage in muscles of mKO mice. On the other hand, increased alanine synthesis and release and persisting peak expression of genes involved in protein degradation suggest that mKO muscle mass should be reduced rather than increased. We thus hypothesized that increased mKO muscle mass must reflect increased rates of daily protein turnover, with net balance favoring muscle protein synthesis. To accurately quantify muscle protein synthesis rates in mKO mice and WT littermates, we used the nonradioactive in vivo surface sensing of translation (IV-SUnSET) technique [78]. We performed experiments at ZT4 and ZT16, in the middle of the physiological fasting and feeding phases, respectively. Muscles from mKO mice showed clearly increased puromycin incorporation into muscle peptides compared to their WT littermates (Fig 7D). Impressively, protein synthesis rates of some mKO mice even approached the extremely high protein synthesis rates observed in a muscle-specific transgenic model with constitutively active AKT (c.a.AKT) [79]. Quantification revealed that WT mice had lower muscle protein synthesis rates at ZT4, the middle of the physiological fasting phase, and approximately 2-fold increased synthesis rates at ZT16, during the middle of the feeding phase (Fig 7E). This is in agreement with previous results obtained in skeletal muscles of fasted and fed WT mice [80,81]. However, muscle protein synthesis rates remained significantly elevated at ZT4 in mKO mice compared to WT littermates.

Increased rates of muscle protein synthesis and increased muscle mass in mKO mice are consistent with functional roles we identified for BMAL1 and REV-ERBα target genes in the regulation of myofiber size and muscle mass (Fig 1B). It is thus probable that these changes in mKO mice arise from local changes in transcription and metabolism inherently linked to loss of BMAL1 rather than changes in feeding behavior or activity. In fact, we could not detect any significant changes in daily feeding pattern or in daily caloric intake, whether measured under standard housing conditions at 22 °C (S4A Fig) or under thermoneutral conditions [82] at 30 °C (Fig 7F). Locomotor activity across the light/dark cycle and total activity levels, measured under thermoneutral conditions (30 °C) by beam breaks (X + Y counts) and running wheel (rotations), were similarly unaltered (Fig 7G).

Differences in body composition are known to impact 24-hr fuel selection and EE [83]. Accordingly, respiratory exchange ratio (RER) measured at 30 °C was significantly lower during the light phase in mKO mice compared to WT littermates, but not during the dark phase (Fig 7H). This is consistent with a shift in nutrient partitioning to promote more fat utilization in mKO animals. Likewise, mKO mice showed significantly increased EE throughout the dark phase at 30 °C (Fig 7I). However, resting metabolic rate (RMR) measured at thermoneutrality throughout the rest+fasting phase [84,85] was not significantly increased in mKO mice, suggesting the approximately 15% increased EE observed during the dark phase reflects a genotype effect rather than differences in body composition. Indeed, even after accounting for differences in lean mass [85], we noted a persisting general trend for increased EE in mKO mice during the dark phase (Fig 7J). A significant genotype effect on total EE (*p* = 0.046) and EE specifically during the dark phase (*p* = 0.023) was confirmed by ANCOVA using body weight, lean mass, and fat mass as covariates, as previously suggested [84].

Overall, these results significantly expand upon our previous observations and indicate that muscle-specific loss of BMAL1 can significantly impact systemic energy homeostasis, alter interorgan metabolite fluxes, and the storage, release, and use of energy substrates in other tissues. Importantly, our cistrome, metabolome, transcriptome, and metabolic phenotyping data all point to fundamental roles of BMAL1 and REV-ERBα in the regulation of energy balance and in systemic lipid and amino acid homeostasis.

### BMAL1 promotes diurnal muscle TG synthesis by direct transcriptional activation of *Dgat2*

**Fig 8:**
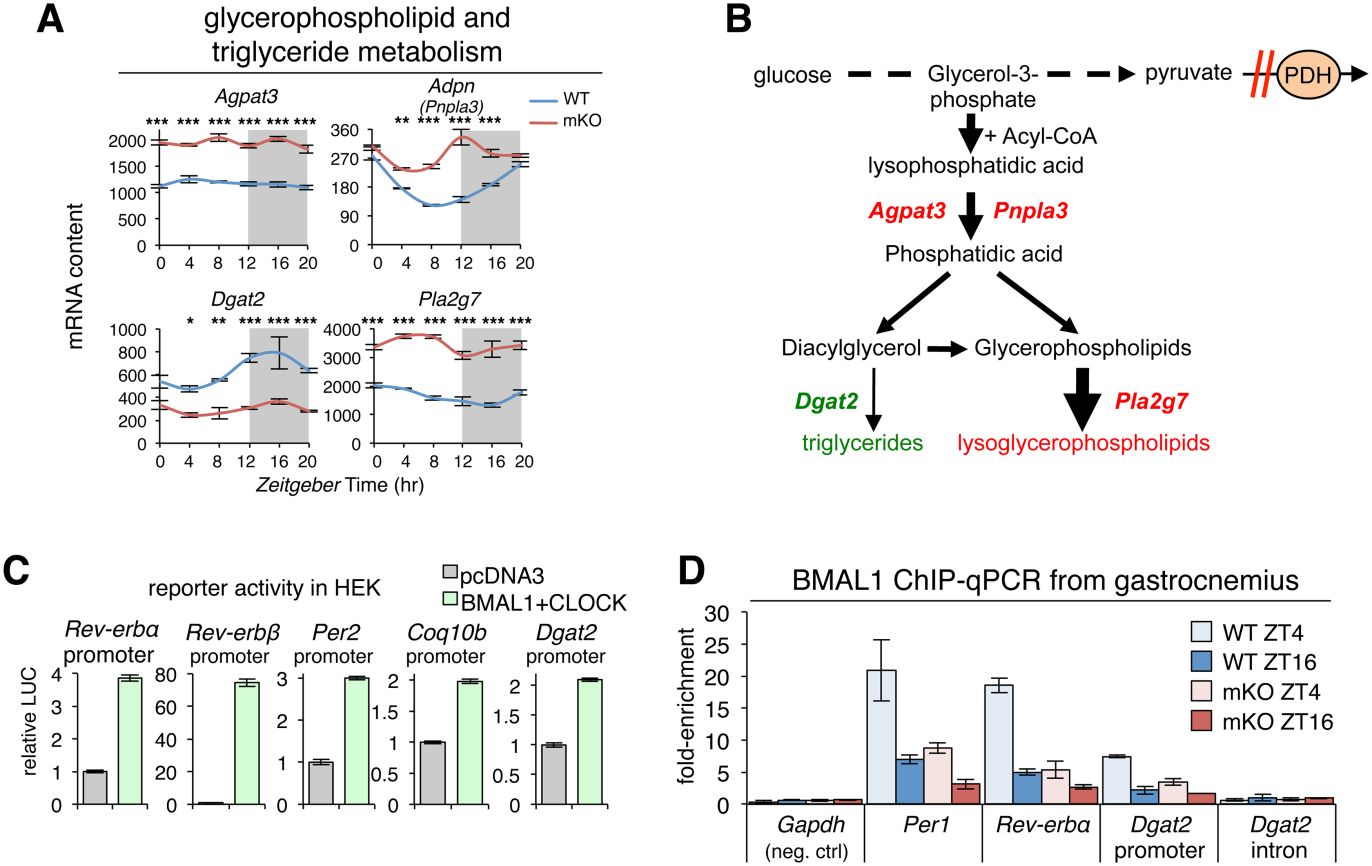
BMAL1 promotes diurnal muscle triglyceride synthesis by direct transcriptional activation of *Dgat2*. (A) Diurnal expression profiles in TA muscles of selected key regulators of glycerophospholipid and triglyceride metabolism (*n* = 3 × time point; mean ± SEM *p < 0.05, **p < 0.01, ***p < 0.001, 2-way ANOVA with Bonferroni correction; see also S7A Fig). (B) Scheme showing alterations identified in mKO muscles related to reduced TG content and increased lysoPLs. Increased glycolytic flux in the context of impaired PDH activity [7] channels glycolytic intermediates to lysoPL biosynthesis. Red = increased gene expression or metabolite abundance in mKO relative to WT; green = reduced gene expression or abundance. (C) Validation of BMAL1 transcriptional targets by cotransfecting HEK-293T cells with LUC reporter constructs containing muscle BMAL1 binding sites from known and putative target promoters linked to LUC, along with expression plasmids for BMAL1 and CLOCK or empty control vector (pcDNA3). Data is expressed as mean fold-change normalized to the empty vector (*n* = 3; ±SEM). (D) In vivo BMAL1 occupancy at target sites in WT and mKO gastrocnemius at ZT4 and ZT16 (mean fold-enrichment over IgG ± SEM; *n* = 2 pooled biological replicates for each of 2 independent ChIP-qPCR experiments). Underlying data can be found in supporting file S1 Data and at Gene Expression Omnibus (accession number GSE43071). BMAL1, brain and muscle ARNT-like protein 1; ChIP-qPCR, chromatin immunoprecipitation–quantitative real-time PCR; CLOCK, circadian locomotor output cycles kaput; HEK-293T, human embryonic kidney 293T; IgG, immunoglobulin G; LUC, luciferase; lysoPL, lysoglycerophospholipid; mKO, myocyte-specific loss of BMAL1; PDH, pyruvate dehydrogenase; TA, tibialis anterior; WT, wild type; ZT, Zeitgeber time.

In addition to its known role driving circadian expression of clock genes like *Rev-erbα/β*, we identified a crucial role for BMAL1 in the regulation of diurnal TG levels. To explain the mechanism behind reduced TG content and increased lysoPLs in mKO muscles, we examined expression profiles of genes involved in glycerophospholipid and TG metabolism. Mutant muscles showed significant differences in expression of 4 crucial enzymes (Fig 8A and S7A Fig). We first noted up-regulation of *Agpat3*, a lysophosphatidic acid acyltransferase abundantly expressed in human skeletal muscle [86] and known PPARα-regulated gene [87]. Likewise, oscillation of *Adpn* (*Pnpla3*), an acyl-CoA-dependent acyltransferase involved in the conversion of lysophosphatidic acid into phosphatidic acid, remained constitutively increased. Phosphatidic acid is a precursor of both TGs and glycerophospholipids, and overexpression of *Adpn* induces both TG and glycerophospholipid synthesis in mammalian cells [88].

Particularly relevant for the reduced TG content in mKO muscles was the marked down-regulation of *Dgat2*, the major enzyme that converts diacylglycerols to TGs in mouse skeletal muscle [89]. According to our muscle cistrome data, *Dgat2* is a direct BMAL1 and REV-ERBα target gene and oscillates with a 24-hr period in both fast and slow muscles [17,90]. In mKO muscles, *Dgat2* oscillation was severely blunted, and expression markedly reduced.

In mammalian cells, glycerophospholipids can be converted to lysoPLs by the activity of specific phospholipases, including phospholipase A2 group 7 (*Pla2g7*), another PPARα-regulated gene that showed markedly increased expression in mKO muscles. Pharmacological activation of PPARα was shown to increase expression of *Pla2g7* and abundance of the LPC(16:0) in serum, liver, and muscle [54]. LPC(16:0) is also an endogenous PPAR ligand that further activates PPARα targets in a feed-forward mechanism. This effect is blocked by *Pla2g7* small interfering RNA (siRNA), and PPARα antagonism also inhibits *Pla2g7* expression.

Modeled together with known perturbations in PDH [7] and diversion of glycolytic intermediates (Fig 8B), our transcript data suggest impaired biosynthesis of TGs yet increased production of bioactive lysoPLs in mKO muscles, in agreement with metabolomics data (Fig 4A–4C). Increased PUFAs in mKO muscles (Fig 4D) is also consistent with increased phospholipase A2 activity, since phospholipase A2 acts specifically at the *sn*-2 position of phospholipids where PUFAs are preferentially located.

To verify whether BMAL1 can transcriptionally activate putative targets identified by our muscle cistromes and transcriptomes, we cotransfected human embryonic kidney 293T (HEK-293T) cells with reporter constructs containing muscle BMAL1 binding sites linked to luciferase, along with empty control vectors or expression plasmids for BMAL1 and CLOCK (Fig 8C). As expected, cotransfection of BMAL1 and CLOCK with reporters of classical known BMAL1 target sites in the promoters of *Rev-erbα*, *Rev-erbβ*, and *Per2* led to a potent induction of transcription (3–70-fold) over basal control levels (empty vector). Likewise, BMAL1 and CLOCK cotransfection with *Coq10b* and *Dgat2* reporters led to a 2-fold transcriptional induction, in agreement with in vivo binding data for BMAL1, and 24-hr *Coq10b* and *Dgat2* expression in WT and *Bmal1* mKO muscles.

To investigate a direct link between loss of genomic BMAL1 binding in mKO muscles and reduced expression and oscillation of putative and established BMAL1 targets, we used directed chromatin immunoprecipitation–quantitative real-time PCR (ChIP-qPCR). We collected mutant and WT muscles at 2 time points, ZT4 and ZT16, i.e., during relatively high and low diurnal genomic Bmal1 binding [11,14]. In WT muscles, we detected specific BMAL1 binding at a positive control locus near *Per1* at ZT4 (Fig 8D) and relatively low binding at ZT16. Importantly, BMAL1 binding was severely attenuated in mKO muscles at both time points. In agreement with muscle-specific loss of *Rev-erbα* expression, BMAL1 binding at a known regulatory locus near *Rev-erbα* was also severely attenuated in mutant muscles. Supporting a direct role in the transcriptional activation of *Dgat2* by BMAL1, we saw similar BMAL1 binding at its target site in the *Dgat2* promoter. Binding was higher at ZT4 in WT muscles yet attenuated in mKO muscles. Furthermore, we detected no BMAL1 binding at a negative intronic *Dgat2* site.

Finally, closer inspection of the *Dgat2* promoter genomic sequence revealed 2 likely tandem E-boxes separated by 15 bp in the very middle of our BMAL1 *Dgat2* peak summit (S7B Fig). Taken together, our data demonstrate that transient binding of muscle BMAL1 to the *Dgat2* promoter is linked to transcriptional activation and a 2–3-fold increase in diurnal *Dgat2* expression. Muscle-specific loss of BMAL1 is associated with 2–3-fold reduced *Dgat2* expression, 2-fold reduced muscle TG content at ZT12, and accumulation of several bioactive lipid species. Muscle-specific overexpression of *Dgat2* was shown to cause a 1.8-fold increase in muscle TG content [91]; thus, modulating local *Dgat2* expression levels is sufficient to increase muscle TG synthesis and storage. Our data highlight mechanistically how BMAL1 promotes diurnal rhythms of neutral lipid storage in preparation for the feeding+activity phase, when bioactive lipids could potentially impair insulin signaling and glucose metabolism. We also show how loss of BMAL1 leads to reduced TG storage and accumulation of bioactive lysoPLs.

### REV-ERBα inhibits lipid and protein catabolism by direct repression of key lipid and protein metabolism target genes

**Fig 9:**
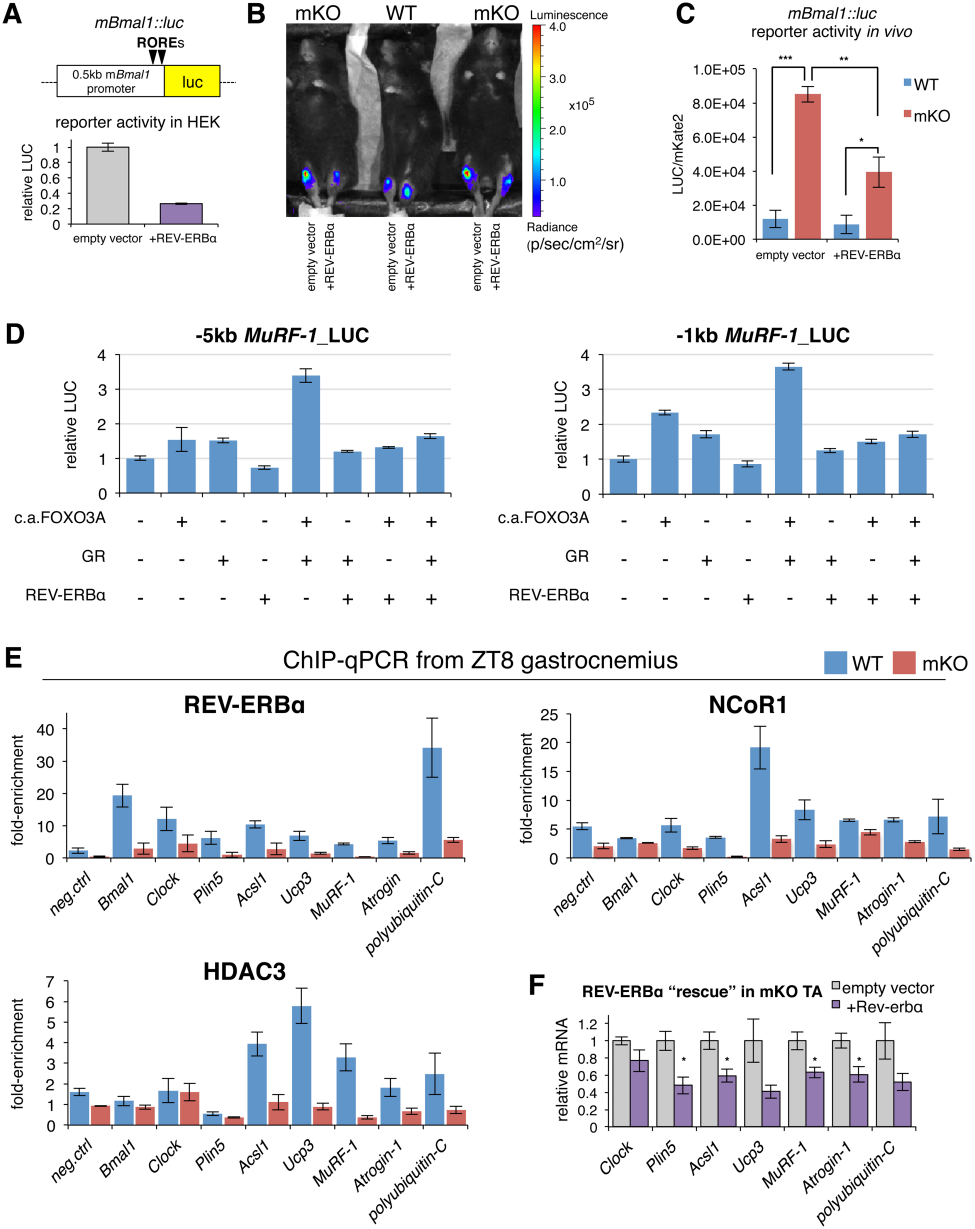
REV-ERBα controls muscle lipid and protein metabolism by directly repressing key regulators. (A) Cotransfection of HEK-293T cells with empty control vector or mouse REV-ERBα expression plasmid, along with REV-ERB sensor (*mBmal1*::*luc*), containing 2 ROREs from the proximal promoter of *Bmal1* linked to LUC. Data from 2 independent experiments are expressed as mean fold-change normalized to the empty vector (*n* = 6; ±SEM). (B) In vivo imaging of LUC activity in WT and mKO mouse TA muscles following electric pulse–mediated gene transfer of *mBmal1*::*luc* cotransfected either with empty vector or REV-ERBα expression plasmid. Imaging analysis was performed 3 d after gene transfer at ZT10 as described in Materials and methods. Pseudocolors overlaid on the image indicate the luminescence intensity from *mBmal1*::*luc* reporter gene activity as indicated by the scale bar radiance (photons/second/cm^2^/steradian). (C) Quantification of LUC activity in TA muscles normalized to mKate2, an exogenous spike-in control plasmid (mean ± SEM; *n* = 3 mice; *p < 0.05, **p < 0.01, ***p < 0.001, Student’s *t* test). (D) Cotransfection of HEK-293T cells with empty control vector, mouse REV-ERBα expression plasmid, mouse GR expression plasmid, or c.a.FOXO3A expression plasmid, along with GR:FOXO sensor containing −5 kb or −1 kb promoter sequence from mouse *MuRF-1* linked to LUC. Data are expressed as mean fold-change normalized to the empty vector (*n* = 2–3; ±SEM). (E) In vivo REV-ERBα, NCoR1, and HDAC3 occupancy at target sites in control and mKO gastrocnemius at ZT8 (mean fold-enrichment over IgG ± SEM; *n* = 4 independent biological replicates from 4 independent ChIP-qPCR experiments; *Foxl2* promoter used as negative control). (F) Gene expression (RT-qPCR) of mKO TA muscles at ZT10 following electric pulse-mediated gene transfer of REV-ERBα expression plasmids or empty vector. Data normalized to *36B4* expression and expressed as fold-change relative to contralateral muscle containing empty vector (mean ± SEM, *n* = 4 mice, *p < 0.05, Student’s *t* test). Underlying data can be found in supporting file S1 Data. BMAL1, brain and muscle ARTN-like protein 1; c.a.FOXO3A, constitutively active forkhead box O3; ChIP-qPCR, chromatin immunoprecipitation–quantitative real-time PCR; CLOCK, circadian locomotor output cycles kaput; GR, glucocorticoid receptor; HDAC3, histone deacetylase 3; HEK-293T, human embryonic kidney 293T; IgG, immunoglobulin G; LUC, luciferase; mKO, myocyte-specific loss of BMAL1; NCor1, nuclear receptor corepressor 1; PDH, pyruvate dehydrogenase; ROR, RAR-related orphan receptor; RORE, ROR response element; RT-qPCR, quantitative reverse transcription PCR; TA, tibialis anterior; ZT, Zeitgeber time.

To monitor in vivo properties of REV-ERB-dependent transcriptional repression in muscles from WT and mKO mice, we used a REV-ERB luciferase reporter (*mBmal1*::*luc*) [92] containing 2 ROR response elements spaced by 26 bp derived from the proximal promoter region of mouse *Bmal1* [93]. Cotransfecting HEK-293T cells with this REV-ERB sensor, along with an expression plasmid for mouse REV-ERBα, led to a 5-fold reduction of transcriptional activity compared to an empty control vector, thus demonstrating its sensitivity to REV-ERBα-mediated repression (Fig 9A). To show that lack of REV-ERBα in mKO muscles is associated with transcriptional derepression of REV-ERBα targets, we used in vivo bioluminescence imaging of TA muscles from WT and mKO mice cotransfected with the REV-ERB sensor and either REV-ERBα or an empty control vector (Fig 9B). Mice were imaged at ZT10, during peak endogenous REV-ERBα and REV-ERBβ protein levels in WT muscles. Accordingly, WT muscles transfected with exogenous REV-ERBα showed only a minor reduction of luciferase activity compared to contralateral muscles transfected with the empty control vector (Fig 9C). Importantly, muscles from mKO mice transfected with the empty vector showed the highest sensor activity, while the introduction (“rescue”) of REV-ERBα in contralateral mKO muscles led to a significant 2-fold repression of luciferase activity. Overall, our data show that lack of REV-ERBα in mKO muscles is associated with increased transcriptional activity (i.e., derepression) of REV-ERBα targets.

Our cistrome data revealed that muscle REV-ERBα peaks are highly enriched with GRE motifs, suggesting binding sites for both factors are within close proximity. Mouse, rat, and human *MuRF-1* all contain a consensus GRE around 200 bp upstream of the transcription start site (TSS) [94], which is directly under the summit of the *MuRF-1* REV-ERBα peak we uncovered. In fact, we noted 2 putative (A/G)GGTCA monomer sites approximately 200 bp and approximately 450 bp downstream of the GRE in the first exon of *MuRF-1* (S8A Fig). A consensus FOXO binding site is also directly adjacent to the GRE, and FOXO and GR were previously shown to synergistically activate *MuRF-1* expression from this same promoter site [94]. To determine the functional relevance of REV-ERBα binding to the *MuRF-1* promoter, we cotransfected luciferase reporter constructs containing either −5 kb or −1 kb fragments of the mouse *MuRF-1* promoter along with expression constructs for mouse REV-ERBα, GR, and a constitutively active form of FOXO3 (c.a.FOXO3A) [95]. GR and c.a.FOXO3A each induced both *MuRF-1* promoter constructs, and they together increased each reporter activity >3-fold (Fig 9D). Addition of REV-ERBα alone or with either GR or c.a.FOXO3A had only a limited repressive effect on either *MuRF-1* reporter; however, REV-ERBα completely blocked the synergistic activation by GR and c.a.FOXO3A when combined together. While several additional interesting muscle REV-ERBα targets suggested by our cistrome and transcriptome data remain to be rigorously validated as functional targets on a case-by-case basis, our results already highlight the role of REV-ERBα repression at major established sites known to be coactivated by GR and FOXO in muscle cells.

To demonstrate that increased expression of putative functional REV-ERBα targets is associated with loss of REV-ERBα genomic binding in mKO muscles, we performed directed ChIP-qPCR of REV-ERBα and corepressors NCoR1 and HDAC3. We focused on selected loci at ZT8, when REV-ERBα is abundant in control muscles (Fig 2C). We detected REV-ERBα binding at positive control loci like *Bmal1* and *Clock* promoters, in addition to binding at novel muscle REV-ERBα loci we uncovered near *Plin5*, *Acsl1*, *Ucp3*, *MuRF-1*, *Atrogin-1*, and *polyubiquitin-C* (Fig 9E). Importantly, binding was severely abrogated in mKO muscles, establishing these as direct REV-ERBα targets. Binding of corepressors NCoR1 and HDAC3 at most sites was likewise reduced in mutant muscles, consistent with loss of REV-ERBα occupancy and increased expression (Fig 2D–2F and S3C Fig).

Finally, to investigate whether REV-ERBα can modulate expression of these genes in vivo, we performed a REV-ERBα “rescue” in mKO muscles via electric pulse–mediated gene transfer. Importantly, the introduction of REV-ERBα into mKO muscles (S8B Fig) led to 20%–50% reduced expression of *Clock*, *Plin5*, *Acsl1*, *Ucp3*, *MuRF-1*, *Atrogin-1*, and *polyubiquitin-C* at ZT10 relative to contralateral muscles transfected with the empty control vector (Fig 9F).

Moving beyond skeletal muscle, we found further evidence for a common network of REV-ERBα-regulated lipid and amino acid metabolism genes in diurnal expression data from liver-specific overexpression of *Rev-erbα* [9]. Since *Bmal1* is a direct target of REV-ERBα, transgenic activation of REV-ERBα constitutively represses *Bmal1* across the light/dark cycle, analogous to our muscle-specific *Bmal1* KO. Likewise, *Rev-erbα* overexpression was linked to reduced expression and blunted diurnal oscillation of *Clock*, *p21* (*Cdkn1a*), *Plin5* (*2310076L09Rik*), and *Snat2* (*Slc38a2*) (S8C Fig).

Altogether, our data highlight a coordinated network of REV-ERBα target genes controlling both lipid and amino acid metabolism in skeletal muscle and perhaps in other tissues. We show how normal diurnal rhythms of muscle REV-ERBα might serve to repress these genes in anticipation of the feeding+activity phase, when glucose returns as the predominant fuel source. Furthermore, muscle-specific loss of BMAL1 also leads to loss of REV-ERBα-dependent repression and persistently increased expression of these targets, likely causing increased lipid metabolism and increased protein turnover.

## Discussion

Here, we present a comprehensive map of in vivo genomic binding for the clock transcription factors BMAL1 and REV-ERBα in adult mouse skeletal muscle and the transcriptional and metabolic consequences of muscle-specific loss of BMAL1 and REV-ERBα. We have sought to shed some light on the complex and highly dynamic regulation of skeletal muscle metabolism from a 24-hr perspective and have attempted to contextualize our results and add functional relevance by linking genomic binding to 24-hr patterns of putative target gene expression and metabolite fluctuations. We hope these rich genomics and metabolomics data profiles will provide a useful resource to others for further hypothesis generation and validation. We have tried to maximize the potential for correlations between different assays by using predominantly fast glycolytic muscles from the same cohort of animals when possible (for example, contralateral TA muscles were used for metabolomics and transcriptomics, gastrocnemius muscles were used for quantitative lipidomics and ChIP-qPCR, vastus lateralis muscles were used for western blotting and mitochondrial respiratory chain activity assays). Details can be found in S4 Table.

**Fig 10:**
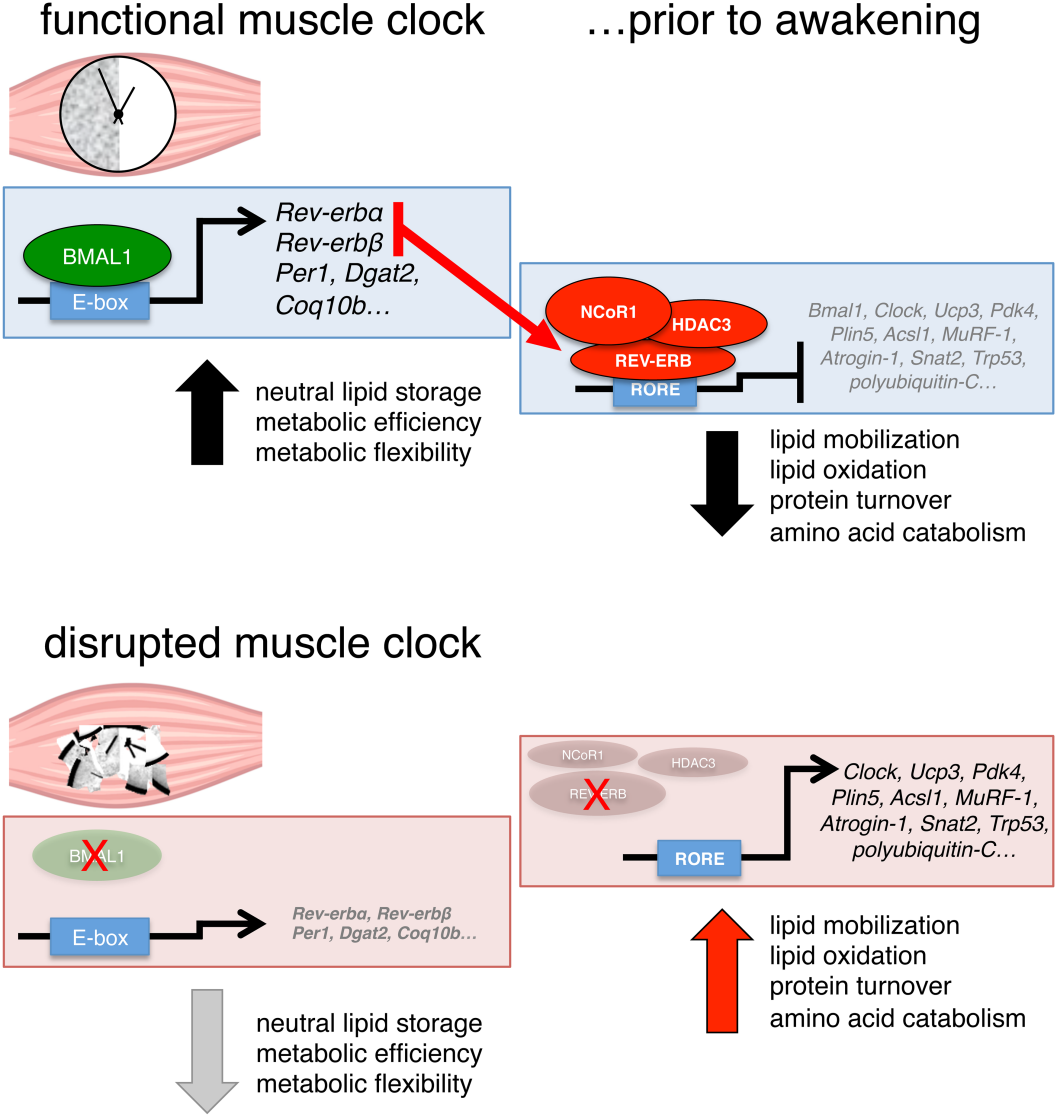
BMAL1- and REV-ERB-dependent programming of muscle metabolism. Our integration of multiple “–omics” datasets indicates that the muscle clock may modulate diurnal fuel selection in anticipation of the feeding phase by direct BMAL1-dependent activation of genes promoting neutral lipid storage (*Dgat2*) and metabolic efficiency (*Coq10b*) while coordinating REV-ERB-mediated repression of a network of genes involved in lipid metabolism (*Plin5*, *Acsl1*, *Ucp3*, *Pdk4*) and muscle protein turnover (*MuRF-1*, *Atrogin-1*, *polyubiquitin-C*, *Snat2*). Muscle clock disruption causes loss of BMAL1-dependent activation and REV-ERB-dependent repression of target genes, resulting in a state of metabolic inefficiency characterized by increased lipid mobilization and oxidation and in increased protein turnover. BMAL1, brain and muscle ARNT-like protein 1; HDAC3, histone deacetylase 3; NCoR1, nuclear receptor corepressor 1; RORE, ROR response element.

To preserve glucose during periods of fasting, peripheral tissue metabolism shifts to prioritize the use of lipids, ketone bodies, and amino acids as energy substrates [96]. This occurs concomitantly with increased peripheral glucose production derived mostly from amino acids supplied from skeletal muscle protein breakdown. Accordingly, low blood glucose and the resulting low circulating insulin levels are catalysts for increased peripheral lipolysis, muscle protein degradation, and amino acid release. These homeostatic adaptations are readily apparent during starvation, high-fat diet, and endurance exercise. Our data suggest that the same mechanisms are at play and relevant during normal 24-hr fasting/feeding and rest/activity cycles. Indeed, the normal circadian rise in blood glucose concentrations at awakening is exquisitely controlled at multiple levels and by multiple tissues to ensure coordinated maintenance of glycemia and peripheral insulin sensitivity [97]. Our study highlights several examples of how the muscle clock plays an important role in these processes by directly modulating and coordinating local transcriptional programs in anticipation of diurnal oscillations of hormones and metabolites (summarized in Fig 10).

Tissue-specific circadian clocks are thought to function as metabolic rheostats [98]. This occurs both by synchronizing cells within each tissue and by fine-tuning local tissue metabolism in anticipation of rhythmic systemic changes in behavior or nutritional state. Our data indicate that the muscle clock is particularly tuned to regulate muscle metabolism, as genomic binding sites for BMAL1 and REV-ERBα were mostly muscle-specific (S1B Fig). This reflects known tissue specificity of circadian clock transcription factor binding [13] and is likely a consequence of differences in tissue-specific chromatin accessibility. Our interpretation is supported by the fact that both factors also shared a muscle-specific enhancer genomic signature, with muscle binding sites for BMAL1 and REV-ERBα also showing high enrichment of motifs for myogenic regulatory factors—like MYOD, MYF5, and MYOG—and coregulators like MEF2.

While common muscle BMAL1 and REV-ERBα target genes largely reflect their involvement in the regulation of glucose metabolism and muscle mass, our REV-ERBα-specific cistrome also highlights a specialized metabolic role in regulating muscle lipid and protein metabolism. This was not previously observed in genome-wide studies mapping REV-ERBα binding in proliferating and differentiating myoblasts in culture [99] and so may be a particular facet of REV-ERBα’s physiological role in differentiated muscle cells within an in vivo context.

Our results suggest that REV-ERBα may regulate lipid and protein metabolism target genes via cross-talk/competition with other relevant nuclear hormone receptors, especially PPARs and GR, but also others, including AR and thyroid hormone receptor (TR). Such promiscuity among nuclear hormone receptors at target sites is well known [100,101] and is thought to permit more subtle or complicated regulatory mechanisms [37]. Highlighting potential sites for cross-talk/competition between REV-ERBα and PPARs, we found REV-ERBα peaks near established [36,37] PPREs of *Plin5* and *Acsl1*. Importantly, overexpression of *Plin5* alone is sufficient to drive expression of a cluster of PPARα target genes involved in lipid catabolism and mitochondrial oxidation in rat TA muscles [102]. This is thought to occur by a feed-forward mechanism whereby increased perilipin-5 (PLIN5) promotes increased production of endogenous PPAR ligands. Increased activation of PPAR targets in mKO muscles may thus likewise result from a loss of REV-ERB-dependent repression of critical targets, including *Plin5*, in conjunction with increased production of endogenous PPAR ligands.

Another potentially important link between rhythmic PPAR activity and the circadian clock is through the PAR-domain basic leucine zipper (PAR bZip) proteins D-site-binding protein (DBP), thyrotroph embryonic factor (TEF), and hepatic leukemia factor (HLF). Like REV-ERBα/β, these transcription factors are direct BMAL1 targets and oscillate with high amplitude over 24 hr at the level of mRNA and protein in most peripheral tissues [103]. They are thought to regulate diurnal liver PPAR signaling indirectly by their transcriptional control of acyl-CoA thioesterase (ACOT) proteins, which in turn liberate fatty acids from acyl-CoA esters that can then serve as endogenous PPARα ligands [104]. Interestingly, *Acot* genes (*Acot1-13*) were generally unchanged or showed slightly increased expression in mKO muscles—despite loss of BMAL1 and despite massively reduced 24-hr oscillation of *Dbp*, *Tef*, and *Hlf*—and increased expression of the PAR bZip repressor *Nfil3* [7], another potential REV-ERBα target gene [105]. Accordingly, it remains to be determined whether there are similar links between PPAR signaling and PAR bZIP factors in muscle.

What is clear from our data is that time of day is a particularly crucial component for physiological REV-ERB action in adult skeletal muscle, as every 12 hr, muscle cells alternate between endogenous REV-ERBα/β gain and loss of function (Fig 2C). These considerations have obvious chronotherapeutic implications for successful pharmacological targeting of muscle REV-ERBα/β. When considering the timing and the relative abundance of REV-ERBα/β protein expression in relation to other nuclear hormone receptors and their known and potential cross-talk, it is very tempting to think of physiological REV-ERB action in terms of a temporal genomic “reset.” In this way, REV-ERBα/β may block and thus resensitize common yet context-dependent regulatory regions of important metabolic target genes, preparing them to respond to the next day’s particular challenges. Future studies are certainly warranted to comprehensively explore and better define these potential relationships.

Our data illuminate how BMAL1, REV-ERBα, and their target genes work together to establish a transcriptional logic that defines 24-hr energy state and a coherent fuel selection within the muscle cell, thus maximizing metabolic efficiency. For example, we show how BMAL1-dependent activation of *Dgat2* could promote synthesis and storage of neutral lipids, while REV-ERB-dependent repression of lipid metabolism genes could coordinately dial down mobilization and oxidation of lipids. This occurs in conjunction with transcriptional activation of gene programs promoting muscle insulin sensitivity and glucose metabolism in anticipation of the feeding+activity phase [7]. In the absence of BMAL1, muscle cells appear to become untethered from (and thus unable to anticipate) rhythmic systemic nutritional signals and persist in a fasting state, resulting in accumulation and de novo diurnal oscillation of lipids and amino acids. Loss of BMAL1 appears to uncouple glycolysis from oxidation, and divert glycolytic intermediates to alternative sugar, nucleotide, lipid, and amino acid biosynthetic pathways (Figs 5F and 8B, and [7]).

Likewise, REV-ERB-dependent inhibition of the ubiquitin-proteasome system (*MuRF-1*, *Atrogin-1*, *polyubiquitin-C*, and proteasome subunits) and the autophagy-lysosome pathway (*Trp53*, *Atg12*, *Mybph*, and others) might provide an anticipatory brake on these metabolically expensive and potentially devastating processes. Our results suggest that BMAL1 thus tempers physiological rhythms of muscle protein breakdown in anticipation of feeding. However, BMAL1’s role in regulating protein turnover appears to be somewhat limited in scope, as it does not play a major role in pathological protein degradation associated with the massively catabolic state of denervation atrophy [106] or in age-associated sarcopenia [3,7]. It will be important to establish whether this is also the case in other forms of muscle atrophy, such as glucocorticoid therapy, sepsis, diabetes, or cancer cachexia. Indeed, one possibility that remains to be tested is whether mKO muscles, or other models of circadian misalignment, exhibit increased sensitivity to endogenous glucocorticoids due to the loss of REV-ERBα-dependent repression. This could lead to the transiently increased expression of GR targets like *MuRF-1*, *Atrogin-1*, *polyubiquitin-C*, and others that we observe.

Reduced peripheral fat mass in mKO mice is in agreement with increased lipid oxidation and reduced lipid storage. On the other hand, increased alanine synthesis and release and persisting peak expression of genes involved in protein degradation suggest that increased mKO muscle mass reflects overall increased protein turnover, with net balance favoring muscle protein synthesis. Indeed, mKO mice showed significantly increased rates of protein synthesis at ZT4 (Fig 7D and 7E). Increased rates of protein synthesis often go hand in hand with increased rates of protein degradation. For example, the paradoxical up-regulation of protein synthesis and mammalian target of rapamycin complex 1 (mTORC1) activation during denervation atrophy occurs via a proteasome-mediated increase in intramuscular amino acids [107]. Likewise, protein degradation is often a necessary correlate of muscle growth/remodeling [108].

One of many interesting REV-ERBα target genes we uncovered was *Snat2* (*Slc38a2*). Sodium-dependent neutral amino acid transporter 2 (SNAT2) is a major determinant of cell size [46] and anabolic amino acid concentration in muscle cells [109] because of its dual role as an amino acid transporter–receptor [110]. SNAT2 couples System A transport with System L activity, thus increasing uptake of BCAA, including the potently anabolic essential amino acid leucine, and activation of mTORC1. Loss of REV-ERB-mediated repression of *Snat2* (Fig 2F and S8C Fig) may thus contribute to the increased BCAA observed at ZT16 in mKO muscles (Fig 5A) and might also contribute to increased rates of muscle protein synthesis. Even a mildly increased futile cycle of protein breakdown and resynthesis in mKO muscles would add a substantial contribution to metabolic rate [111,112] and negatively impact overall metabolic efficiency. Indeed, we also noted significantly increased EE in mKO mice (Fig 7I).

Our finding of increased protein synthesis in mKO muscles appears to contrast with the reported cytosolic role of BMAL1 as a translation factor that promotes rhythmic protein synthesis as a substrate of the mTOR-effector p70S6K1 (S6K1) [113]. Lipton and colleagues found approximately 10% reduced in vivo protein synthesis rates in livers from WT mice between ZT0 and ZT12, the beginning and the end of the fasting phase, respectively. On the other hand, livers from global BMAL1 KO mice showed approximately 30% reduced protein synthesis rates at ZT0 and ZT12 compared to WT mice. Examining different timepoints, in a different tissue, and using a different in vivo model of BMAL1 ablation, we found approximately 2-fold-increased protein synthesis rates in WT muscles at ZT16, the middle of the feeding+activity phase, compared to WT muscles at ZT4, the middle of the fasting+rest phase. While seemingly at odds, our results and those of Lipton and colleagues in WT mice are both in agreement with published results obtained from livers and muscles of fasted and fed WT mice [80]. However, we found that protein synthesis rates remained elevated rather than reduced in mKO muscles at ZT4. Some important considerations may explain possible discrepancies. For example, liver protein synthesis rates are much higher than skeletal muscle under fed/fasted states and over 24 hr [114,115]. Furthermore, muscle and liver show important tissue-specific differences in their regulation of protein synthesis [116]. For example, rapamycin-induced inhibition of mTORC1 completely blocks the stimulation of protein synthesis in liver upon feeding yet only partially inhibits muscle protein synthesis [117]. In addition, leucine increases protein synthesis in skeletal muscle but not in liver [118–121]. Finally, mice lacking S6K1 have normal global translational activity in muscle [115] and continue to show increased muscle protein synthesis and hypertrophy in response to AKT activation [122].

The uptake of oxidative substrates and their rate of flux through catabolic pathways are ultimately determined by the efficiency of electron transport and proton conductance (the sum of ATP synthesis and proton leak) [69]. Increased oxidative capacity in conjunction with increased uncoupling from ATP production is seen in endurance-trained athletes [72] and in rodents fed a high-fat diet [74]. The resulting loss of metabolic efficiency (i.e., uncoupling substrate oxidation from ATP production) is thought to protect against an excessive buildup of Δ*ψ*_m_ and uncontrolled formation of reactive oxygen species (ROS) [68]. Diurnal mitochondrial coupling efficiency is thus likely lowest from ZT8 to ZT12, at the end of the resting phase, characterized by low respiration rates, low levels of intracellular ADP (S5B Fig), and around peak diurnal expression of *Ucp3* and *Ucp2* (Fig 6G). In this state, total oxygen consumption would be dominated by basal uncoupling mechanisms [72], and decreased efficiency of energy production would cause a concomitant rise in basal TCA cycle flux. In WT muscles, ZT8–ZT12 coincides with the peak of diurnal citrate levels, which oscillate around 2–3-fold over 24 hr. Increased TCA flux at this time may be beneficial in that it could provide a more rapid response in anticipation of increased energy demand upon awakening, analogous to its advantageous role proposed in endurance athletes compared to sedentary controls [72]. However, despite increased muscle TCA intermediates, there is no clear advantage gained in terms of exercise endurance in mKO mice (S6B Fig), and so increased TCA cycle anaplerosis may simply reflect a mismatch between pyruvate formation and oxidation, as suggested by Gibala and colleagues [59].

It is important to stress that the mild mitochondrial alterations we observe in mKO muscles should not be considered in terms of dysfunction. Interestingly, our results are in stark contrast to the severe exercise intolerance and mitochondrial dysfunction reported for *Clock* mutant mice [123] and global *Rev-erbα* KO mice [29]. However, interpretation of these particular global models and their relation to the muscle clock is complicated by the fact that all tissues are affected, including the central pacemaker. Further studies using conditional muscle-specific and inducible *Clock* and *Rev-erbα/β* KO models, also keeping in mind REV-ERBα’s indirect DNA binding roles [13], would certainly help to clarify the particular relationships. Rather, our data suggest the mitochondrial alterations we observe in mKO muscles are likely operating within a normal physiological range, albeit at one extreme analogous to the fasted state. While loss of BMAL1 leads to a mild state of metabolic inefficiency under chow diet and standard housing conditions, endogenous muscle antioxidant systems appear to sufficiently buffer any associated increase in oxidative stress.

Our data thus support the idea that specifically inhibiting muscle REV-ERBα/β [124] in certain situations may provide a relatively safe means to temporarily modulate metabolic efficiency and EE in skeletal muscle, a major oxidative tissue and relevant pharmacological target in humans [125,126]. Indeed, pioneering groups have already begun to wade into these therapeutic waters and, importantly, have already shown in mice that selective REV-ERB antagonism accelerates muscle regeneration in response to acute muscle injury [99] and promotes muscle differentiation and maintenance of muscle mass in a model of Duchenne muscular dystrophy (DMD) [127]. Intriguingly, Welch and colleagues showed REV-ERB antagonism enhances Wnt and Notch signaling, leading to satellite cell self-renewal. Selective inhibition of REV-ERBα/β in muscle would also promote increased *Bmal1* expression, which is proposed to have positive effects on sleep [128] and longevity [129], and would further promote neutral lipid storage (Figs 4A and 8), muscle insulin sensitivity, and glucose metabolism [7].

In conclusion, we present a comprehensive in vivo view of BMAL1 and REV-ERBα genomic binding in adult mouse skeletal muscle and show how this can be related to common and specific transcriptional programs and metabolite oscillations in the presence or absence of muscle BMAL1 and REV-ERBα/β. Our integration and presentation of multiple in vivo high-throughput “–omics” datasets provides an entry point to explore how BMAL1 and REV-ERBα can direct various physiological processes, including the regulation of muscle energy homeostasis. Our data suggest that the muscle clock, via BMAL1 and REV-ERBα, plays an important role regulating metabolic efficiency by (1) confining substrate use to appropriate temporal windows, (2) modulating rates of energetically expensive protein turnover, (3) promoting storage of neutral lipids, and (4) controlling oxidation of lipid substrates. We also show how the muscle clock may specifically inhibit a network of genes involved in lipid mobilization and oxidation, protein degradation, and amino acid transport via direct REV-ERB-mediated repression in anticipation of the feeding phase, all while coordinately activating genes promoting neutral lipid storage, insulin sensitivity, and glucose oxidation [7]. The translational relevance of our findings is already evident based on a series of very elegant recent studies performed by Hansen [130], Loizides-Mangold [131], Perrin [132,133], van Moorsel [134], and colleagues using human biopsies and human muscle cells in culture. While we have focused only on 2 circadian clock transcription factors and a small subset of potential target genes, additional mediators and physiological roles of the muscle clock remain to be explored. We hope the comprehensive nature of our resource will serve as an important basis from which to continue the exploration of muscle circadian rhythms in health and disease.

## Materials and methods

### Contact for reagent and resource sharing

Further information and requests for resources and reagents should be directed to and will be fulfilled by Kenneth Dyar (kenneth.dyar@helmholtz-muenchen.de).

### Ethics statement

All experimental procedures were performed according to European Commission guidelines and were reviewed and approved by the local Veterinary Central Service, University of Padova, and the relevant Italian authority (Ministero della Salute, Ufficio VI), in compliance with Italian Animal Welfare Law (Law n 116/1992 and subsequent modifications) and Directive 2010/63/EU of the European Parliament. Accordingly, experiments performed before 2012 were sent to the Italian Ministry of Health (Project# 27/09). Experiments performed after 2012 were approved by the Italian Ministry of Health (Decree# 164/2012-B of 09.08.12).

### Animals

Animals were housed in a temperature-controlled room (22 °C) under a 12-hr light/dark regimen, with lights on at ZT0 (6 AM) and lights off at ZT12 (6 PM), with standard chow diet (Mucedola, Settimo Milanese, Italy) and water provided ad libitum. Muscle-specific inactivation of *Bmal1* (mKO) was obtained as described [7] by crossing a floxed *Bmal1* C57BL/6 mouse line with a C57BL/6 mouse line carrying a Cre recombinase transgene under control of the *Mlc1f* promoter (*Mlc1f-Cre*). In the resulting mKO mice, the region coding for the BMAL1 bHLH DNA binding domain is excised. Cre-negative littermates were used as controls. All mice used in this study were 3–5-mo-old male littermates, unless specified otherwise. Littermates were randomly assigned to experimental groups. Tissues were collected immediately after cervical dislocation at ZT0, 4, 8, 12, 16, and 20, snap frozen in liquid nitrogen, and stored at −80 °C until subsequent use. S4 Table details the various muscles and tissues used for this paper.

### Metabolic phenotyping

Total body fat and lean tissue mass were quantified by nuclear magnetic resonance (EchoMRI). EE, RER (VCO2/VO2), locomotor activity, and feeding behavior were monitored on a TSE system (Bad Homburg, Germany).

### Exercise and endurance training experiments

To measure exercise endurance, forced running on a motorized treadmill was used (Harvard Apparatus PANLAB, LE 8710 M). The protocol was based on [135], with some minor modifications. Briefly, mice were first acclimated to low-speed running (22 cm/s and 10% incline) for 10 min each day for 2 d prior to the test. Acclimation was always performed under dim red light around ZT12 (lights off), the normal start of the circadian activity phase. On the day of the endurance test, food was removed 3 hr before starting at ZT12 to ensure consistent baseline blood glucose levels. For the test, mice began running at 22 cm/s and 10% incline under dim red light. Speed was gradually increased by 5 cm/s every 30 min up to 45 cm/s. Mice were encouraged to run by humanely stimulating their tail with a soft brush. The operator was blind to genotype of the mice, and exhaustion was defined by mice refusing to run for 10 sec and confirmed by blood glucose <76 g/dl [75]. Time and running distance were recorded for each mouse. For endurance training, mice trotted on a motorized treadmill 25 cm/s and 0% incline 1 hr daily for 4 wk, starting around ZT12. After humanely encouraging mice to run with a soft brush on their tail during the first few acclimation sessions, mice ran without much need for further encouragement.

### Clinical blood chemistry

Sedentary mice were either fed ad libitum, and blood was collected at ZT14 (“fed ZT14”), or fasted for 4 hr late in the afternoon (ZT7–ZT11), and blood was collected at ZT11 (“fasted ZT11”). A separate cohort of endurance-trained WT and mKO littermates was measured after 4 wk training on a motorized treadmill 25 cm/s and 0% incline 1 hr daily, starting around ZT12. Blood was collected after running 25 cm/s and 0% incline for 1 hr. Blood was collected from the orbital sinus in heparin-coated Pasteur pipettes and centrifuged immediately after collection. Plasma samples were kept at −20 °C until dosing. FFAs, β-OH-B, and AcAc were dosed using an automated spectrophotometer, Cobas Fara II (Roche), according to the manufacturer’s instructions. Blood glucose and lactate levels were measured with a YSI 2300 STAT Plus glucose and lactate analyzer (YSI Life Sciences, Yellow Springs, OH) according to the manufacturer’s instructions.

### Quantification of serum amino acids

Mice were fasted 6 hr (8 AM–2 PM), and blood was collected at ZT8 (2 PM). Blood was collected from the orbital sinus in Pasteur pipettes, allowed to clot at room temperature (RT) for 30 min, centrifuged, and kept at −20 °C until use. Amino acid concentrations were assessed by GC/MS (HP 5890; Agilent Technologies, Santa Clara, CA), using the internal standard technique, as previously reported [136]. Briefly, known amounts of internal standards (L-[^15^N]glycine, L-[^15^N]glutamate, L-[^15^N]glutamine, L-[1-^13^C, methyl-^2^H_3_]methionine, L-[3,3-^2^H_2_]cysteine, L-[^15^N]alanine, L-[1-^13^C]leucine, L-[1-^13^C]phenylalanine, L-[3,3-^2^H_2_]tyrosine, L-[^15^N]threonine, L-[^15^N]serine, and L-[^15^N]proline [Cambridge Isotope Laboratories]) were added to plasma samples. Amino acid concentrations were determined considering the following mass-to-charge ratios (m/z): 218/219 for glycine, 432/433 for glutamate, 431/432 for glutamine, 320/324 for methionine, 406/408 for cysteine, 158/159 for alanine, 302/303 for leucine, 336/337 for phenylalanine, 466/468 for tyrosine, 404/405 for threonine, 362/363 for serine, and 184/185 for proline.

### In vivo ChIP assays

In vivo skeletal muscle ChIP was performed with sonicated nuclear extract prepared from formaldehyde-cross-linked gastrocnemius muscle according to [137]. For immunoprecipitation, we used anti-BMAL1 (ab93806, Abcam), anti-REV-ERBα (generous gift from Ron Evans), anti-RNA Polymerase II (#MMS-126R; clone 8WG1, Biolegends), anti-NCOR1 (#20018-1-AP, Protein Tech), anti-HDAC3 (ab7030, Abcam), or rabbit IgG (2027x, Santa Cruz). DNA was column purified and used for sequencing or real-time qPCR (enrichment expressed as fold-change relative to IgG; primer sequences used are listed in S4 Table).

### ChIP-seq library prep

Libraries from ChIP and input DNA were prepared with the KAPA Hyperprep Kit (Kapa Biosystems, KK8504); Illumina-compatible adapters were synthesized by Integrated DNA Technologies (IDT) and used at a final concentration of 68 nM. Adapter-ligated libraries were size selected (360–610 bp) in a Pippin Gel station (Sage Science) using 2% dye free gels (Sage Science, CDF2010). Library concentration was estimated by real-time PCR with the KAPA Library Quantification Kit (Kapa Biosystems, KK4873). Library quality was evaluated with the Agilent High Sensitivity DNA Kit on a 2100 Bioanalyzer (Agilent). Libraries were run on a HighSeq2500/HighSeq4000 sequencers (Illumina) at the NGS Core Facility at HMGU.

### Bioinformatics pipeline for ChIP-Seq data analysis

#### Pre-processing

ChIP-Seq FASTQ files were mapped against the mouse mm10 genome with BWA version 7.12 using MEM algorithm [138]. Duplicate reads were removed using samtools version 0.1.19 [139]. Multi-mapping reads were removed with bamtools version 2.4.0 [140] using read threshold of MAPQ ≥ 24.

#### Adjusting sequencing depth

Sequencing read depth was adjusted by down sampling replicate BAM files to the replicate with lowest read count, resulting in about 13 million unique reads for RNAP2 and about 20 million unique reads each for BMAL1 and REV-ERBα replicates (see Table 1 below for further details).

**Table 1:** Counts of samples at different stages of bioinformatics analysis.

sample description	map percent	read #	rmdup read #	unique reads # (MAPQ > 24)	downsampled unique reads #	# peaks *p* < 0.05	high confidence peaks
Muscle_RNAP2_ZT4_rep1	96.75	18,912,373	16,083,495	12,951,866	12,951,866	87,115	} 10764
Muscle_RNAP2_ZT4_rep2	99.33	26,923,018	23,744,982	21,078,107	13,000,000	168,711	
Muscle_Bmal1_ZT4_rep1	99.15	27,754,186	21,676,362	19,181,165	19,181,165	34,730	} 2787
Muscle_Bmal1_ZT4_rep2	97.85	44,673,573	32,176,505	28,279,965	20,000,000	177,586	
Muscle_Rev-erbα_ZT8_rep1	99.68	36,031,011	28,439,234	25,292,094	20,000,000	28,832	**} 1263**
Muscle_Rev-erbα_ZT8_rep2	99.43	28,909,625	22,233,385	19,698,054	19,698,054	29,883	
Muscle_Input_rep1	99.11	28,600,958	26,311,286	23,540,615			
Muscle_Input_rep2	99.58	29,104,973	24,849,024	21,920,901			

Abbreviation: MAPQ, mapping quality.

#### Peak calling

Macs2 version 2.1.1 [141] peak caller was used to perform peak calling on the replicates using input DNA. Peak calling cutoff was set to *p*-value 0.05. In addition to narrow peak files, read density distribution (Bedgraphs) was used to visualize ChIP-seq tracks using Integrated Genome Browser [142].

#### Peak universe, “high confidence” and “confident” peak tables, and overlapping peaks

A peak table was created for each sample. Replicate sample tables were then combined into a unified peak universe for each factor with unique ranges across the genome and containing overlapping peaks information including the tag counts (enrichment score). “High confidence” peaks were identified as reproducible peaks common between replicate samples. “Confident” peaks were reproducible peaks common between BMAL1 and REV-ERBα and present in ≥3 of the 4 samples.

To assess overlapping peaks between liver and muscle, liver peak location coordinates for BMAL1 [14] and REV-ERBα [15] were first converted from mm9 to mm10 using UCSC liftOver.

#### Heatmaps

Bedgraphs were used to plot the read density map near the universe peak centers using deepTools version 2.2.4 [143]. Replicates for each factor were merged using UCSC tools bedGraphToBigWig and bigWigMerge (http://hgdownload.cse.ucsc.edu/admin/exe/macOSX.x86_64/).

Then, deepTools computeMatrix tool was used followed by plotHeatmap tool [143].

#### Peak annotation

Peak annotation was performed with HOMER version v4.8 [144]. GENCODE database for mm10 [145] was used as a reference for assigning feature level annotation.

#### Motif discovery

To minimize bias, a combination of two different methods was used for motif discovery:

1. HOMER version v4.8 [144]. For BMAL1 we searched for motifs of 8-, 10-, 12-, 14-, 15-, and 16-mers. For REV-ERBα, we searched for motifs of 8-, 10-, and 12-mers.
2. Clover version published on Jan 14, 2016 [146], was used with default settings.

#### Functional enrichment analysis of peaks

Genomic Regions Enrichment of Annotations Tool (GREAT) [16] was used to analyze functional significance of BMAL1 and REV-ERBα peaks using mouse NCBI build 38 genome assembly (mm10), whole genome as background, and associating genomic regions with genes using “basal plus extension” and the following parameters: proximal 20 kb upstream, 2 kb downstream, plus distal up to 500 kb.

### Global metabolite profiling

Metabolite profiling, peak identification, and curation were performed by Metabolon using described methods [147]. Briefly, the nontargeted metabolic profiling platform used by Metabolon combines 3 independent platforms: UHPLC/MS/MS optimized for basic species, UHPLC/MS/MS optimized for acidic species, and GC/MS. We analyzed a total of 60 TA muscles from 60 male muscle-specific *Bmal1* KO and control littermates (5 × group × time point).

### Metabolomics data processing and analysis

Metabolomics data (“origscale”) can be found in supporting file S3 Data. The data were first normalized according to raw area counts and processed according to [148]. Run day correction was performed for each metabolite by setting the run day medians equal to 1. We removed metabolites with more than 50% missing values and transformed data to log10. Data points outside 4 times the standard deviation for each metabolite were considered as outliers and removed. Missing data were imputed by k-nearest-neighbor algorithm. To identify metabolites that show significant change over time and/or genotype, we fit data to a linear mixed effects model. Significant changes were estimated by performing F-test statistics to each fixed effect term (ANOVA) Genotype, Time, Genotype × Time. Calculations were done using MATLAB R2015b, Statistics Toolbox. Heatmaps were generated using the mean of 5 replicates. Hierarchical clustering was performed with squared euclidean distance and Ward’s minimum variance algorithm. Data were sorted by phase according to WT muscle and aligned between groups to show effect in mKO. Metabolites were categorized according to Metabolon superpathways: Amino Acids, Carbohydrates, Cofactors and Vitamins, Energy, Lipids, Nucleotides, Peptides, and Xenobiotics. To identify significantly enriched KEGG pathways in our data, we performed a hypergeometric distribution test on metabolites showing a Genotype effect *p* < 0.05. To identify 24-hr cycling metabolites, we used the nonparametric test JTK_CYCLE as described in [147], using an adjusted *p* < 0.05.

### Extraction of total lipids

Muscles were weighed and homogenized in 800 μl dH2O. Lipids were extracted twice according to Folch and colleagues [149] using chloroform/methanol/water (2/1/0.6, v/v/v) containing 500 pmol butylated hydroxytoluene, 1% acetic acid, and 100 pmol of internal standards (ISTD, 17:0–17:0 PC, 17:0 LPC, 17:0–17:0–17:0 TG, Avanti Polar Lipids) per sample. Extraction was performed under constant shaking for 90 min at RT. After centrifugation at 1,000 x *g* for 15 min at RT, the lower organic phase was collected. Then, 2.5 ml chloroform was added to the remaining aqueous phase, and the second extraction was performed as described above. Combined organic phases of the double extraction were dried under a stream of nitrogen and resolved in 900 μl 2-propanol/chloroform/methanol (7/2/1, v/v/v).

### Quantitative analysis of TG by HPLC with light scattering detection

For HPLC-ELSD, 20 μl of the resolved extract was evaporated and dissolved in 100 μl chloroform/methanol (2/1, v,v). The chromatographic setup for lipid separation consisted of an Agilent 1100 combining pump, injector, precooled sample manager (4 °C), and column oven (40 °C) (Agilent, Santa Clara, CA). For detection of lipids, a Sedex 85 evaporative light scattering detector (Sedere, Alfortville, France) was used. Data acquisition was performed by the Chemstation software (B 04.01, Agilent, Santa Clara, CA). A ternary gradient with a Betasil Diol column (100 × 4.6 mm, particle size 5 μm, Thermo Fisher Scientific, Waltham, MA) was used for chromatographic separation. The solvent system consisted of eluent A (isooctane/ethylacetate, 99.8/0.2, v/v), eluent B (acetone/ethylacetate, 2/1, v/v, containing 0.02% [v/v] acetic acid), and eluent C (isopropanol/water, 85/15, v/v, containing 0.05% [v/v] acetic acid and 0.3% [v/v] ammonium acetate). For external calibration, TG 54:3 (Larodan, Solna, Sweden) was prepared in chloroform:methanol (2:1, v/v), and the final concentration ranged from 1 μM to 2.5 μM. Injection volume for all samples including external calibration was 10 μl.

### Qualitative analysis of lipids by ultra-performance liquid chromatography (UPLC) with qTOF detection

For UPLC-qTOF, 120 μl of the resolved extract was transferred to an autosampler vial for analysis. Chromatographic separation was performed using an AQUITY UPLC system (Waters), equipped with a BEH-C18-column (2.1 × 150 mm, 1.7 μm; Waters) as previously described [150]. A SYNAPTG1 qTOF HD mass spectrometer (Waters) equipped with an ESI source was used for detection. For positive and negative ionization mode, 5 μl and 10 μl were injected, respectively. Data acquisition was done by the MassLynx 4.1 software (Waters Corporation). Lipid classes were analyzed with the “Lipid Data Analyzer 1.6.2” software [151]. Extraction efficacy and lipid recovery were normalized using ISTD, and lipid classes were expressed as percent composition.

### Separation of neutral lipids by thin-layer chromatography

Extracted lipids were spotted on a silica gel 60 (Merck, Darmstadt, Germany). For comparison, a standard solution containing TG 54:3 was used. The silica gel was developed using n-hexane/diethylether/acetic acid (80/20/2, v/v/v) as solvent system, and lipids were visualized by charring using concentrated sulfuric acid.

### Palmitate oxidation

Gastrocnemius muscles were immediately removed after cervical dislocation. Tissues were quickly weighed, and placed in ice-cold homogenization buffer (250 mM Sucrose, 10 mM Tris-HCL, 1 mM EDTA, pH = 7.4). Muscles were then thoroughly minced with scissors and transferred to a 10 ml glass homogenization mortar on ice, and ice-cold homogenization buffer was added up to a 20-fold dilution (w/v) suspension. Samples were then homogenized with 10 strokes of a motor-driven, tightly fitting glass mortar/Teflon pestle Potter Elvehjem homogenizer operated at 1,600 rpm. Reactions were initiated by adding 50 μl of muscle homogenates to 450 μl of prewarmed oxidation medium (30 °C; 111 mM sucrose, 11.1 mM Tris-HCl, 5.56 mM KH_2_PO_4_, 1.11 mM MgCl_2_, 88.9 mM KCl, 0.222 mM EDTA, 1.11 mM DTT, 2.22 mM ATP, 0.33% fatty acid–free BSA, 2.22 mM Carnitine, 0.056 mM CoA, 0.111 mM Malate, 222 uM cold Palmitate, + 0.5 uCi [1-^14^C]-palmitic acid; prepared fresh daily; pH 7.4). After incubation for 90 min in a shaking water bath (100 rpm; 30 °C), reactions were terminated by addition of 100 μl 1 M perchloric acid, and the CO2 produced during the incubation was trapped in 100 μl NaOH that had been added to a small tube inside the reaction vial. Palmitate oxidation rates were determined by measuring incorporation into ^14^CO_2_ and ^14^C-acid-soluble metabolites (ASM) by liquid scintillation counting.

### Analysis of mitochondrial function

Spectrophotometric activity of mitochondrial respiratory chain complexes CI, CII, CIII, CII+III, CIV, as well as CS, was measured according to [152] using muscle homogenates from frozen vastus lateralis muscles from the same cohort of mice used for gene expression and metabolomics analyses. Mitochondrial membrane potential was measured by epifluorescence microscopy based on the accumulation of TMRM fluorescence in isolated muscle fibers from flexor digitorum brevis (FDB) muscles as previously described [153], with minor modifications. Briefly, fresh FDB muscles from adult mice were digested in type I collagenase at 37 °C for 2 hr and dissociated into single fibers by gentle pipetting. Isolated FDB myofibers were then placed in 1 ml Tyrode’s buffer (Sigma) and loaded with 2.5 nM TMRM (Molecular Probes) supplemented with 1 μM cyclosporine H (a P-glycoprotein inhibitor) for 30 min at 37 °C. At the times indicated by arrows, oligomycin (Olm, 5 μM; Sigma) or the protonophore FCCP (4 μM; Sigma) was added to the culture medium.

### Assessment of muscle protein synthesis

Quantification of muscle protein synthesis rates was performed using the nonradioactive IV-SUnSET technique as described in [78].

### Gene expression profiling and analyses

Microarray sample processing, quality control, and data analysis are reported in [7].

### SDS-PAGE and western blotting

Muscle lysates were prepared in RIPA buffer (Sigma) supplemented with Halt protease and phosphatase inhibitor cocktail (#78446, Thermo Scientific), protein concentration determined using the bicinchoninic acid assay (Pierce, Rockford, IL, USA), and SDS-PAGE performed using NuPAGE 4%–12% gels (Invitrogen). Proteins were transferred onto a PVDF membrane and incubated with rabbit anti-REV-ERBα (kind gift from Ron Evans), mouse anti-REV-ERBβ (D-8; sc-398252, Santa Cruz), or mouse anti-GAPDH (ab8245, Abcam). Membranes were then incubated with HRP-conjugated donkey anti-rabbit (sc-2317, Santa Cruz) or goat anti-mouse (170–6516, Biorad) secondary antibodies. HRP activity was measured by chemiluminescence (Immobilon Western, Millipore), and bands visualized on CP-BU Medical X-Ray Film (Agfa HealthCare NV, Gevaert, Belgium).

### Plasmids

Mouse promoter regions of *Rev-erbα* (forward, 5′-CCCCTAGTCACCACTAACCTC-3′; reverse, 5′-AGAGACGTGTGCCCTGCTA-3′), *Rev-erbβ* (forward, 5′-ATGTAGGAGGGAGGCTCGG-3′; reverse, 5′-GCCTCGCGCAGACTATGG-3′), *Dgat2* (forward, 5′-AGCTGCTAGGATTGTAGGATTACAG-3′; reverse, 5′-AGAGCTGAGGTAGGTAGCCG-3′), and *Coq10b* (forward, 5′-GCTAACCAAATGCAGCAGGC-3′; reverse, 5′-TGTGAAGCCGGTAGCCAAC-3′) were amplified using High Fidelity Platinum Taq DNA polymerase (Invitrogen), cloned into pGL3-Basic (Promega), and verified by sequencing. pRL-CMV renilla expression construct was from Promega. pCMV-AC-mKate (mKate2) was from OriGene. Both *mPer2*::*luc* (423-bp *mPer2* promoter fragment) and *mBmal1*::*luc* (530-bp *mBmal1* promoter fragment) reporter plasmids were generous gifts from Kazuhiro Yagita and described in [154]. Mouse full-length *Bmal1* and *Clock* pcDNA3-HA constructs were generous gifts from Marina Antoch (Roswell Park Cancer Institute) and described in [155]. Mouse full-length *Rev-erbα* ORF (forward, 5′-ATGACGACCCTGGACTCCAA-3′; reverse, 5′-TCACTGGGCGTCCACCCGGA-3′) was amplified using AccuPrime GC-Rich DNA Polymerase (Invitrogen) and shuttled into Gateway pDONR-221 (Invitrogen) with Gateway BP Clonase Enzyme (Invitrogen). Mouse *Rev-erbα* ORF was then shuttled into Gateway pcDNA-Dest47 expression vector with Gateway LR Clonase Enzyme (Invitrogen). The −1 kb and −5 kb *MuRF-1* promoter luciferase reporters and c.a.FOXO3A plasmids were generous gifts from Marco Sandri. The mouse GR construct was generated by PCR amplifying a Kpn1/BamH1 PCR fragment from full-length mouse GR ORF (BioScience IMAGE40111802) using forward 5′-GGGGTACCATGGACTCCAAAGAATC-3′ and reverse 5′- CGGGATCCTCATTTCTGATGAAAC-3′ primers and cloning into Kpn1/BamH1-digested pcDNA3.

### In vitro transfection and luciferase assays

HEK-293T cells were cultured in DMEM with 10% FBS and penicillin-streptomycin. Cells were seeded in 96-well plates at 100,000 cell/mL for transfection and luciferase measurements. For BMAL1 target validation, cells were cotransfected with 25 ng promoter reporter construct, 25 ng pRL-CMV, and either 25 ng BMAL1-HA + 25 ng CLOCK-HA or 50 ng empty pcDNA3. For REV-ERBα sensor validation, cells were cotransfected with 25 ng *mBmal1*::*luc*, 25 ng pRL-CMV, and either 200 ng REV-ERBα or 200 ng empty pcDNA-Dest47. For MuRF-1 reporter experiments, cells were cotransfected with 25 ng *MuRF-1* reporter, 25 ng pRL-CMV, and 25 ng each of GR, c.a.FOXO3A, and REV-ERBα. To maintain consistent DNA transfection concentrations across experiments, 50 ng or 25 ng empty pcDNA-Dest47 was supplemented, respectively, when transfecting only one or two transcription factors. Transfection was performed with FuGene HD (Promega) and transfection medium replaced with Phenol Red free DMEM. The next day, cells were lysed, and firefly and renilla luciferase were sequentially measured using Dual-Glo Luciferase assay system (Promega). Firefly raw values were normalized to Renilla raw values for each replicate. Data from 2 independent experiments are expressed as mean fold-change of the test condition normalized to the empty vector ± SEM.

### In vivo transfection of adult skeletal muscle

Six-mo-old adult male *Bmal1* mKO mice and WT littermates were anesthetized by i.p. injection of a mixture of Zoletil 100 (a combination of Zolazapam and Tiletamine, 1:1, 10 mg/kg, Laboratoire Virbac) and Rompun (Xilazine 2%, 0.06 ml/kg, Bayer). Gene transfer of TA muscles was induced by intramuscular injection of plasmid DNA (40 μg, consisting of 15 μg *mBmal1*::*luc* reporter plasmid, 5 μg mKate2 exogenous spike-in control, and 20 μg either REV-ERBα or empty pcDNA-Dest47) followed by electroporation using stainless steel electrodes connected to a ECM830 BTX porator (Genetronics, San Diego, CA).

### Optical bioluminescence imaging

In vivo bioluminescence images were acquired with the IVIS 100 system (Perkin-Elmer) under general anesthesia by i.p. injection of a mixture of Zoletil 100 (a combination of Zolazapam and Tiletamine, 1:1, 10 mg/kg, Laboratoire Virbac) and Rompun (Xilazine 2%, 0.06 ml/kg, Bayer) and analysis performed according to [156] with the following parameters: field of view 25 cm, binning factor 8, exposure time 1 min. Living Image software (version 4.3) was used for image capture and analysis.

### RNA isolation and qPCR

Total RNA was isolated, purified, and reverse transcribed to cDNA, and RT-qPCR was performed as described in [7]. Analysis was performed using the standard curve method, and all data were normalized relative to *36B4* expression.

### Quantification and statistical analysis

All data are expressed as means ± SEM unless otherwise stated. Statistical analysis was performed using unpaired Student’s *t* test or 2-way ANOVA. When ANOVA revealed significant genotype differences, further analysis was performed using Bonferroni’s multiple comparison test. Differences between groups were considered statistically significant for *p* < 0.05.

## Supporting information

S1 Data Numerical data behind figures. (XLSX)

S2 Data Motif location and frequency data. (XLSX)

S3 Data Raw 24-hr metabolomics data. (XLSX)

S1 Fig Supporting information for Fig 1. (TIF)

S2 Fig Supporting information for Fig 1. (TIF)

S3 Fig Supporting information for Fig 2. (TIF)

S4 Fig Supporting information for Fig 4. (TIF)

S5 Fig Supporting information for Fig 5. (TIF)

S6 Fig Supporting information for Fig 6. (TIF)

S7 Fig Supporting information for Fig 8. (TIF)

S8 Fig Supporting information for Fig 9. (TIF)

S1 Table Cistrome supporting data. (XLSX)

S2 Table Motif enrichment analysis. (XLSX)

S3 Table ANOVA analysis of metabolomics data. (XLSX)

S4 Table Reagents and resource descriptions. (XLSX)

## Abbreviations

3PG: 3-phosphoglycerate
5’-GMP: 5’-guanosine monophosphate
5’-IMP: 5’-inosine monophosphate
AcAc: acetoacetate
ACOT: acyl-CoA thioesterase
AMP: adenosine monophosphate
AR: androgen receptor
ARE: androgen response element
ASM: ^14^C-acid-soluble metabolites
β-OH-B: β-hydroxybutyrate
BCAA: branched-chain amino acid
bHLH: basic helix-loop-helix
BMAL1: brain and muscle ARNT-like protein 1
c.a.AKT: constitutively active AKT
c.a.FOXO3A: constitutively active forkhead box O3
ChIP-qPCR: chromatin immunoprecipitation–quantitative real-time PCR
ChIP-seq: chromatin immunoprecipitation followed by next-generation sequencing
CLOCK: circadian locomotor output cycles kaput
CS: citrate synthase
DBP: D-site-binding protein
DMD: Duchenne muscular dystrophy
DR1: direct repeat 1
DR2: direct repeat 2
EE: energy expenditure
FCCP: carbonylcyanide-p-trifluoromethoxyphenyl hydrazone
FDB: flexor digitorum brevis
FDR: false discovery rate
FFA: free fatty acid
FOXO: forkhead box O
GC/MS: gas chromatography/mass spectrometry
GDP: guanosine diphosphate
GO: Gene Ontology
GR: glucocorticoid receptor
GRE: glucocorticoid response element
GREAT: Genomic Regions Enrichment of Annotations Tool
GSSG: oxidized glutathione
GTP: guanosine triphosphate
HDAC3: histone deacetylase 3
HEK-293T: human embryonic kidney 293T
HLF: hepatic leukemia factor
IDT: Integrated DNA Technologies
IR3: inverted repeat 3
IV-SUnSET: in vivo surface sensing of translation
KO: knockout
LC/MS: liquid chromatography/mass spectrometry
LPC: lysophosphatidylcholine
lysoPC: lysoglycerophosphocholine
lysoPE: lysoglycerophosphoethanolamine
lysoPI: lysoglycerophosphoinositol
lysoPL: lysoglycerophospholipid
MAPK: mitogen-activated protein kinase
MEF2: myocyte enhancer binding factor 2
MGI: Mouse Genome Informatics
mKO: myocyte-specific loss of BMAL1
MSigDB: Molecular Signatures Database
mTORC1: mammalian target of rapamycin complex 1
MYF5: myogenic factor 5
MYOD: myoblast determination protein
MYOG: myogenin
n.s.: not significant
NCoR1: nuclear receptor corepressor
NEFA: non-esterified fatty acid
NFκB: nuclear factor kappa B
NPAS2: neuronal PAS domain protein 2
PAR bZip: PAR-domain basic leucine zipper
PDH: pyruvate dehydrogenase
*Pla2g7*: phospholipase A2 group 7
PLIN5: perilipin-5
PPAR: peroxisome proliferator–activated receptor
PPRE: PPAR regulatory element
PUFA: polyunsaturated fatty acid
RER: respiratory exchange ratio
RMR: resting metabolic rate
ROR: RAR-related orphan receptor
RORE: ROR response element
ROS: reactive oxygen species
RT: room temperature
RXR: retinoid X receptor
S6K1: p70S6K1
SNAT2: sodium-dependent neutral amino acid transporter 2
TA: tibialis anterior
TCA: tricarboxylic acid
TEF: thyrotroph embryonic factor
TG: triglyceride
TGFβ: transforming growth factor beta
THRα: thyroid hormone receptor alpha
TR: thyroid hormone receptor
TSS: transcription start site
UPLC: ultra-performance liquid chromatography
WT: wild type
ZT: Zeitgeber time
