# Correction: Transcriptional programming of lipid and amino acid metabolism by the skeletal muscle circadian clock

**DOI:** 10.1371/journal.pbio.3000035

**Published:** 2018-09-26

**Authors:** The PLOS Biology Staff

## Notice of Republication

This article was republished on September 7, 2018, to correct the article type to "Methods and Resources". The publisher apologizes for this error. The scientific content of the article has not changed.

## Supporting information

S1 File Originally published, uncorrected article. (PDF)

S2 File Republished, corrected article. (PDF)

## References

[1] Dyar KA, Hubert MJ, Mir AA, Ciciliot S, Lutter D, et al (2018) Transcriptional programming of lipid and amino acid metabolism by the skeletal muscle circadian clock. PLoS Biology 16(8): e2005886 10.1371/journal.pbio.2005886 30096135 PMC6105032

