# Supplementary figures and images for: Transcriptional programming of lipid and amino acid metabolism by the skeletal muscle circadian clock
Source: PLoS Biol. 2018 Aug 10;16(8):e2005886. doi: 10.1371/journal.pbio.2005886 (PMC6105032; doi:10.1371/journal.pbio.2005886)

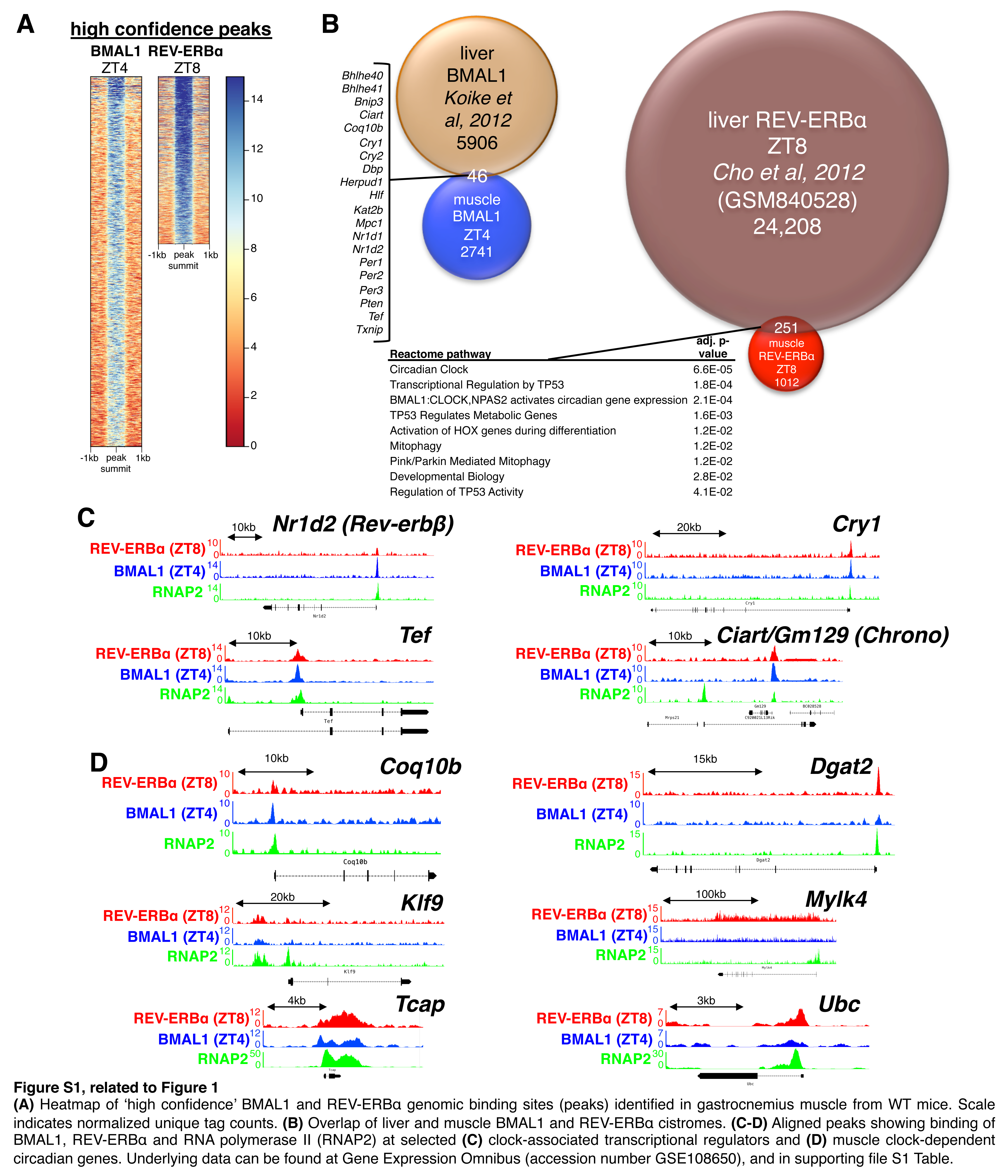

Supplement: S1 Fig — (TIF) [file pbio.2005886.s004.tif]

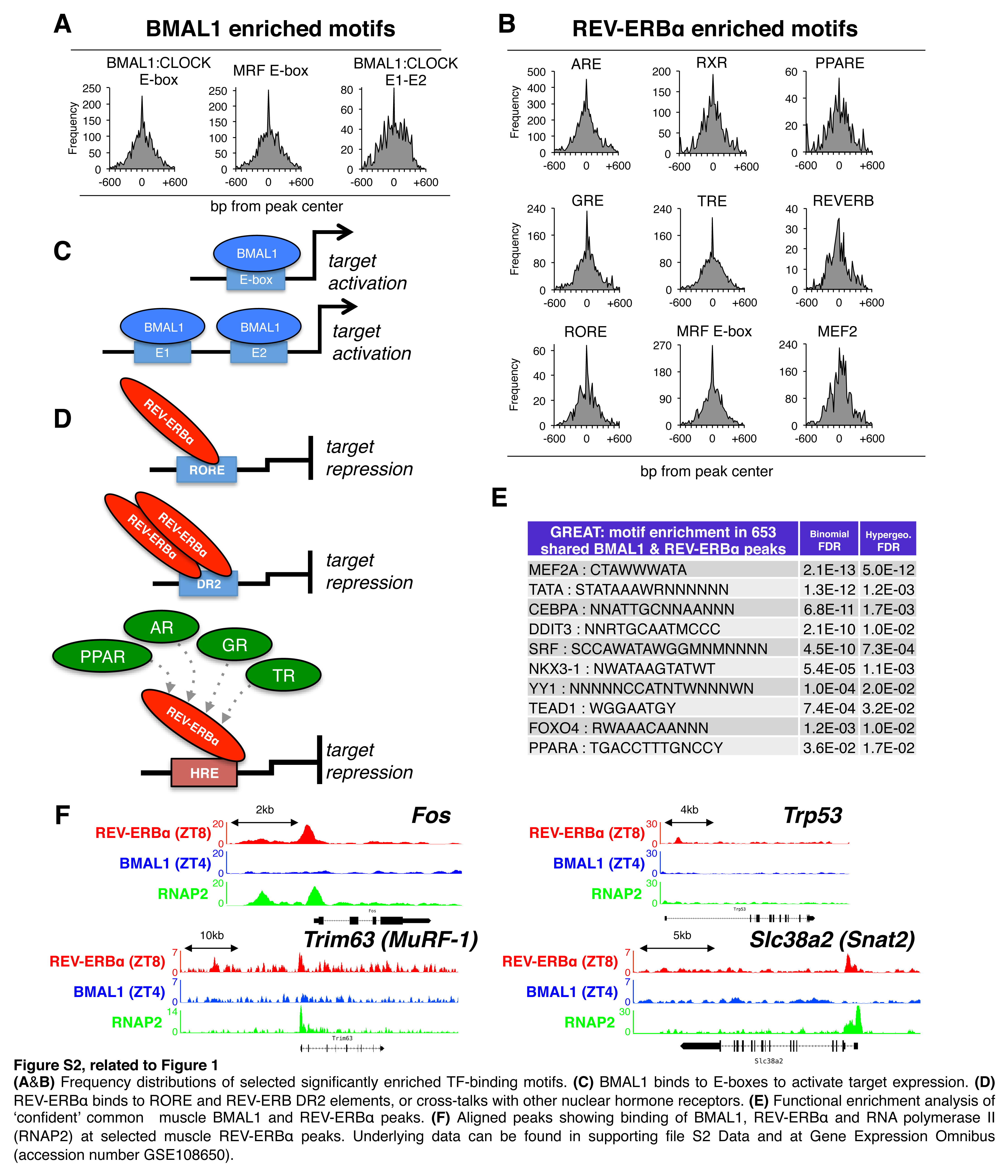

Supplement: S2 Fig — (TIF) [file pbio.2005886.s005.tif]

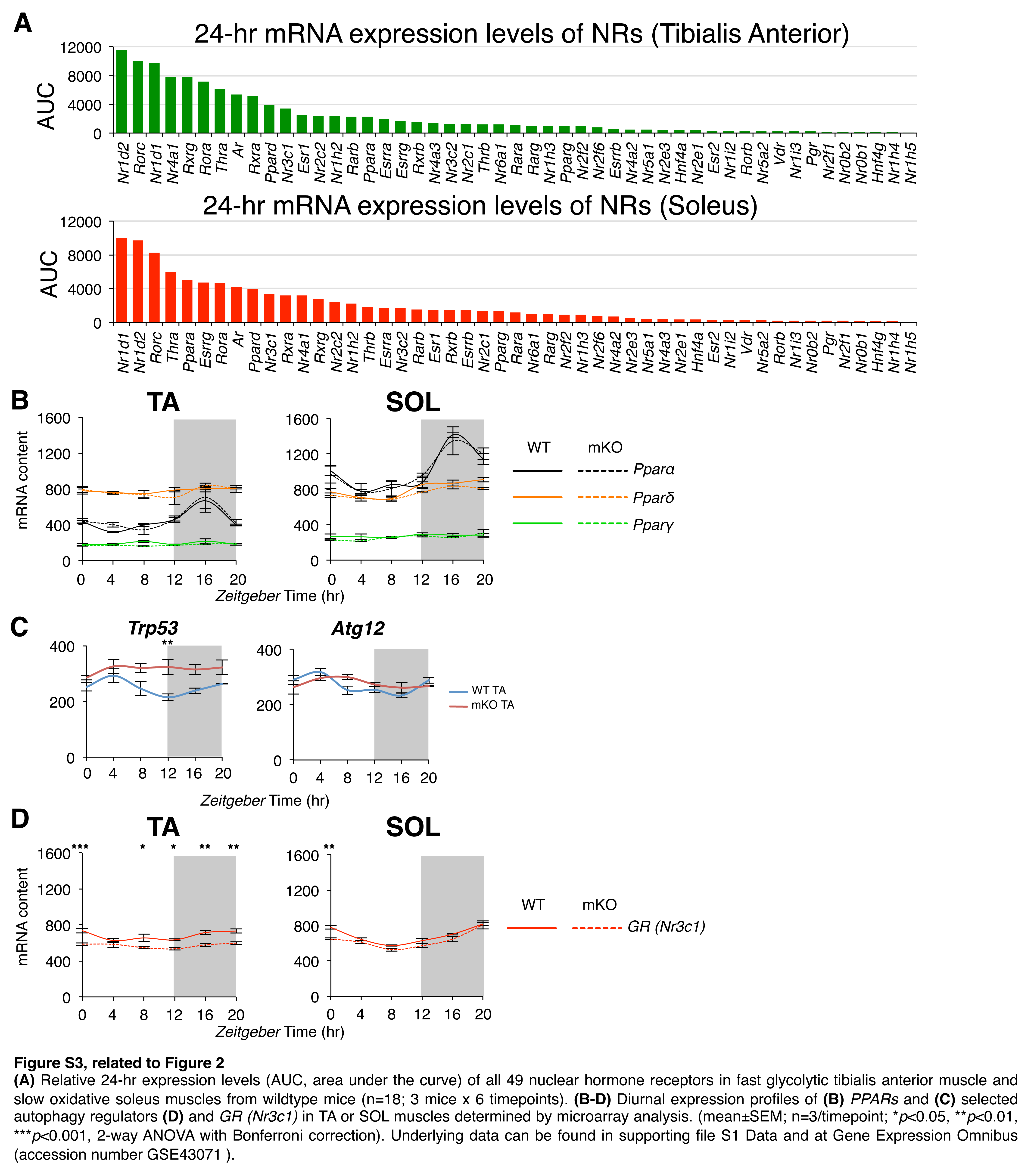

Supplement: S3 Fig — (TIF) [file pbio.2005886.s006.tif]

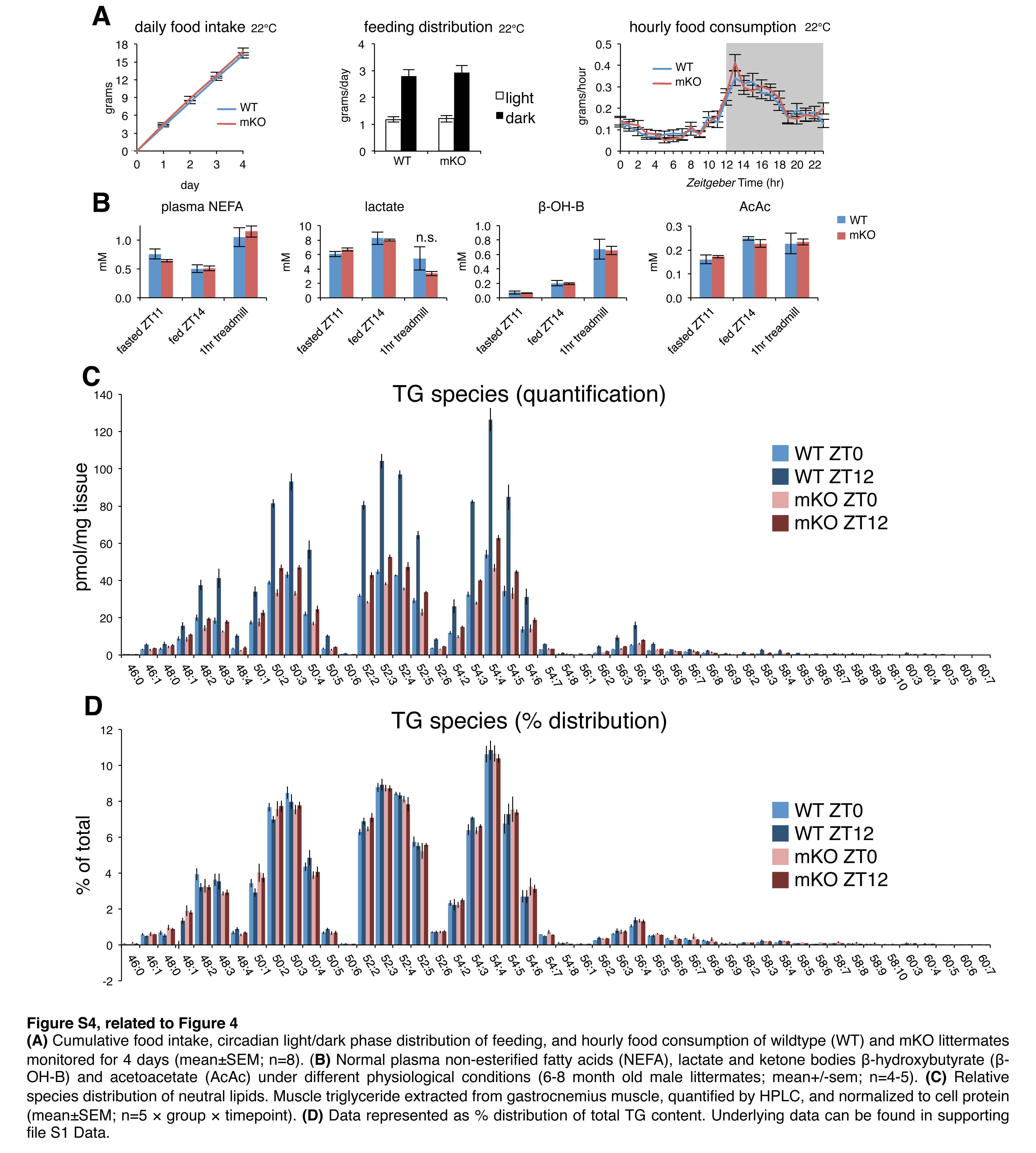

Supplement: S4 Fig — (TIF) [file pbio.2005886.s007.tif]

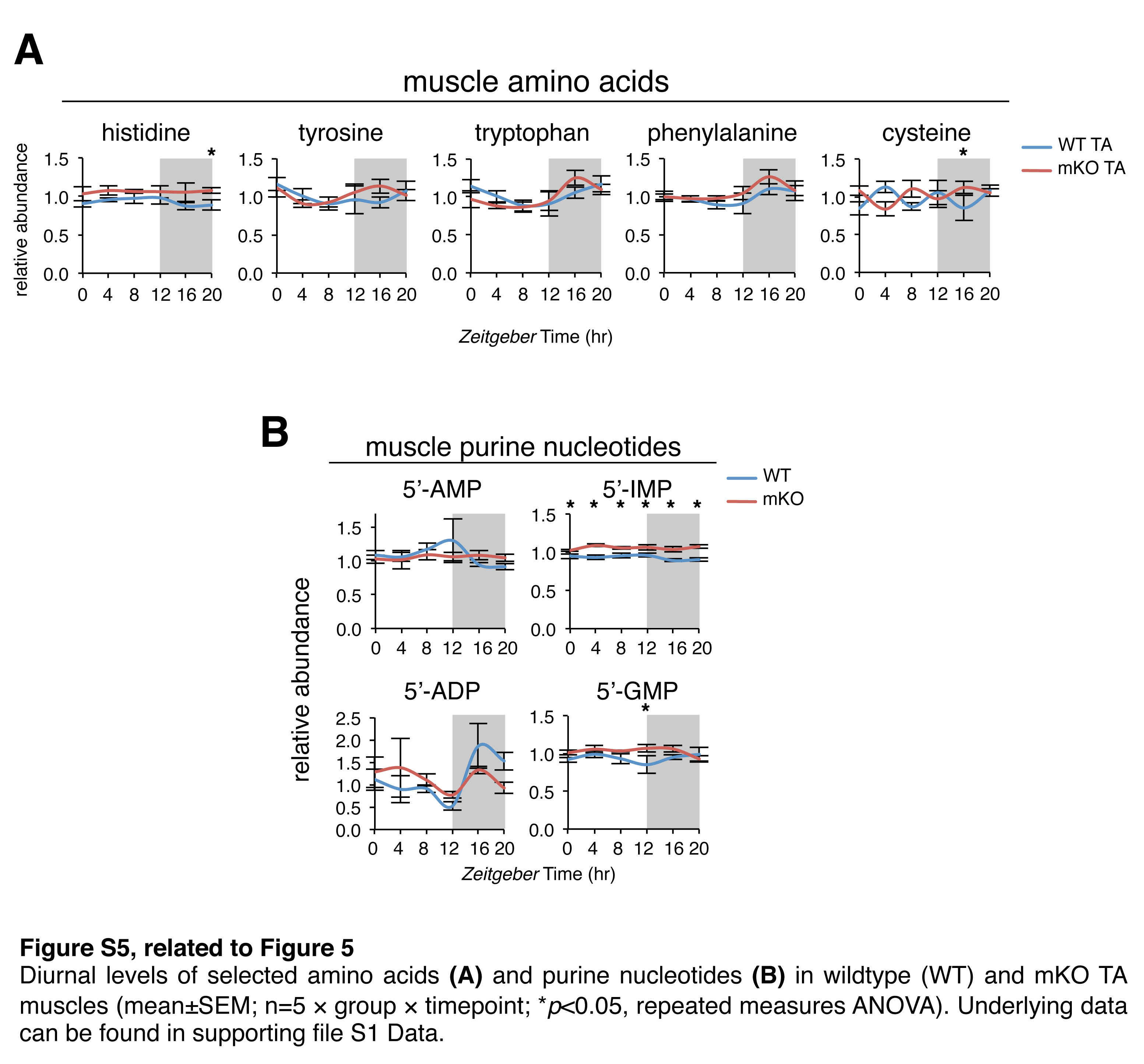

Supplement: S5 Fig — (TIF) [file pbio.2005886.s008.tif]

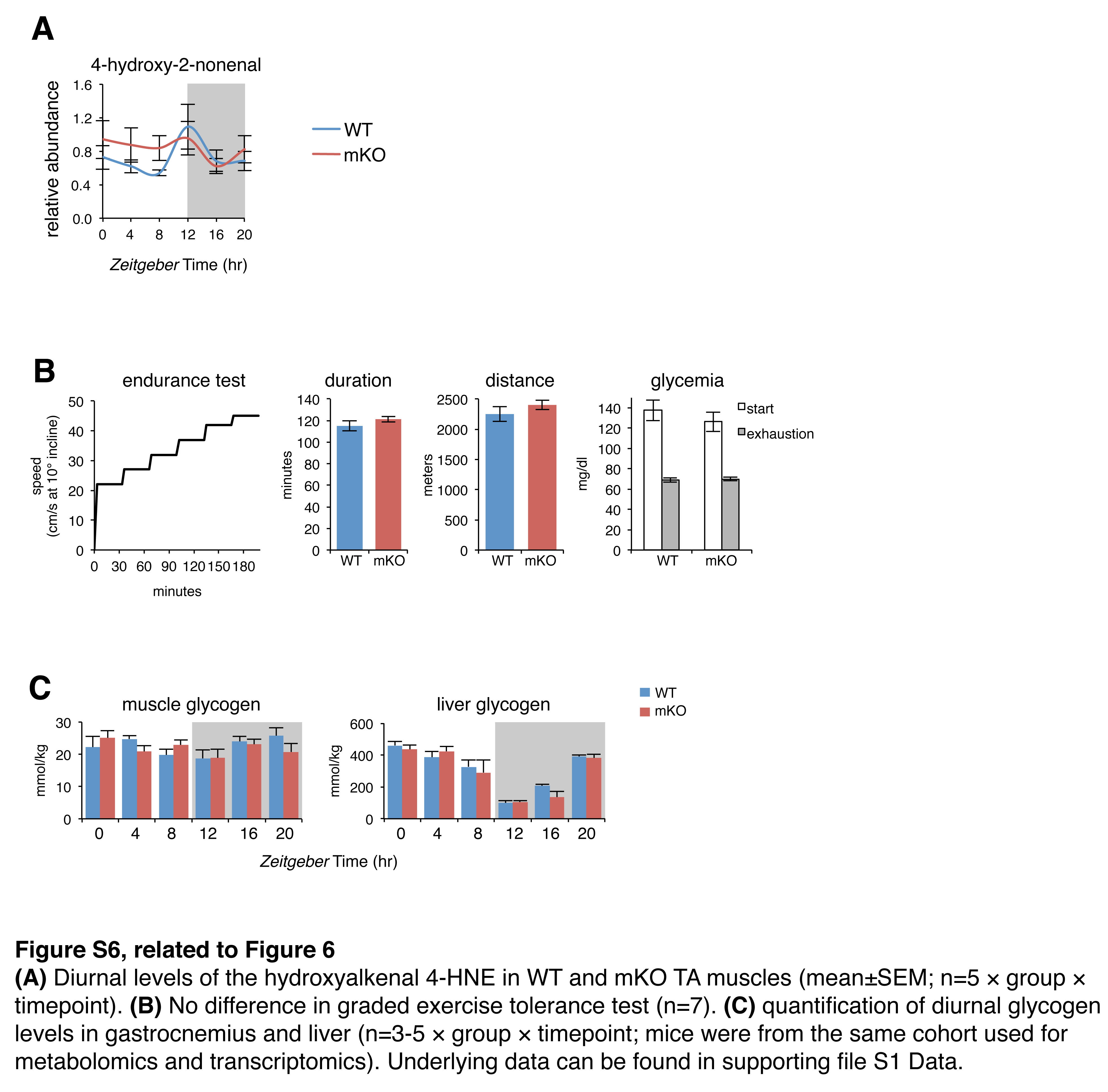

Supplement: S6 Fig — (TIF) [file pbio.2005886.s009.tif]

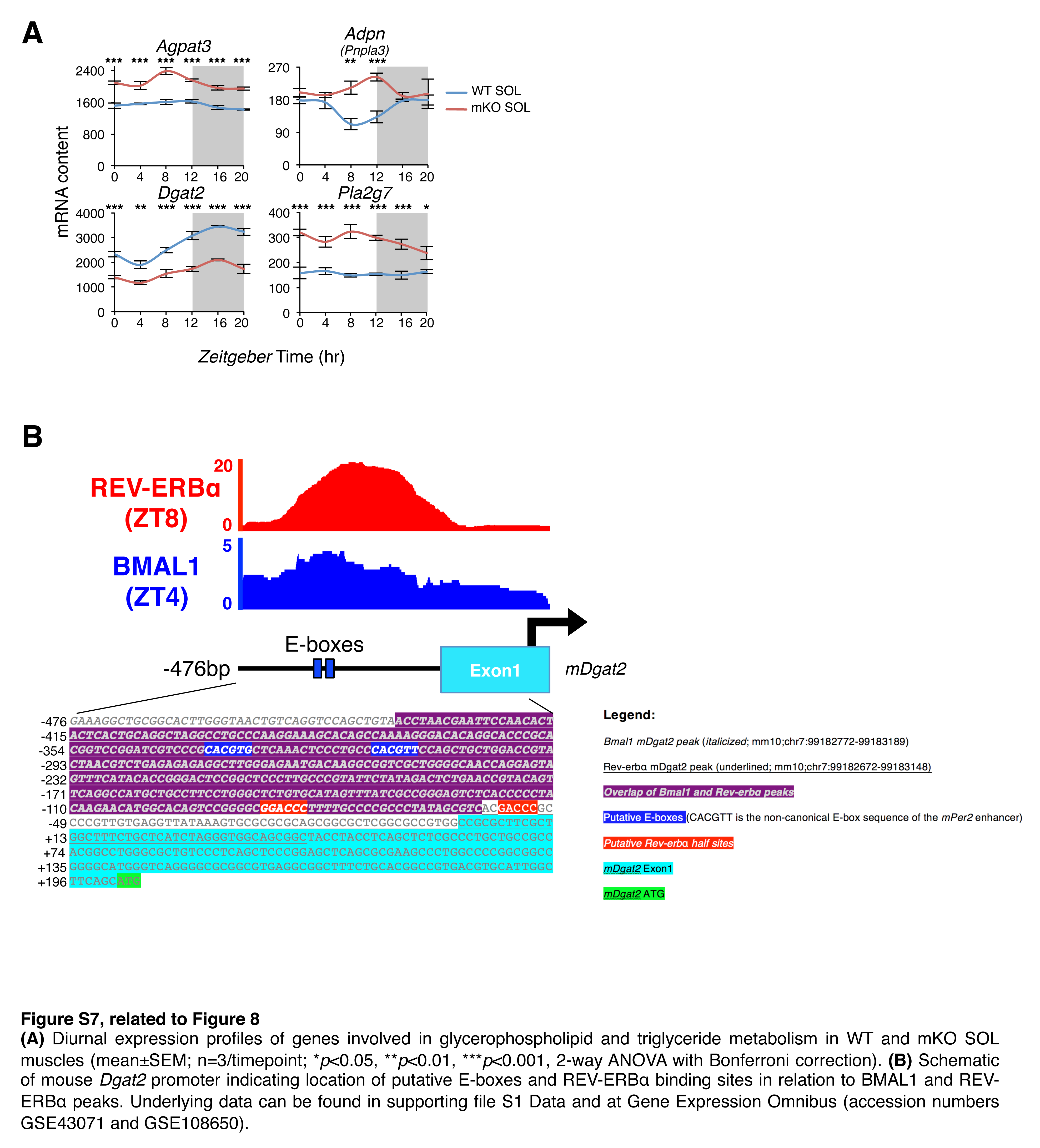

Supplement: S7 Fig — (TIF) [file pbio.2005886.s010.tif]

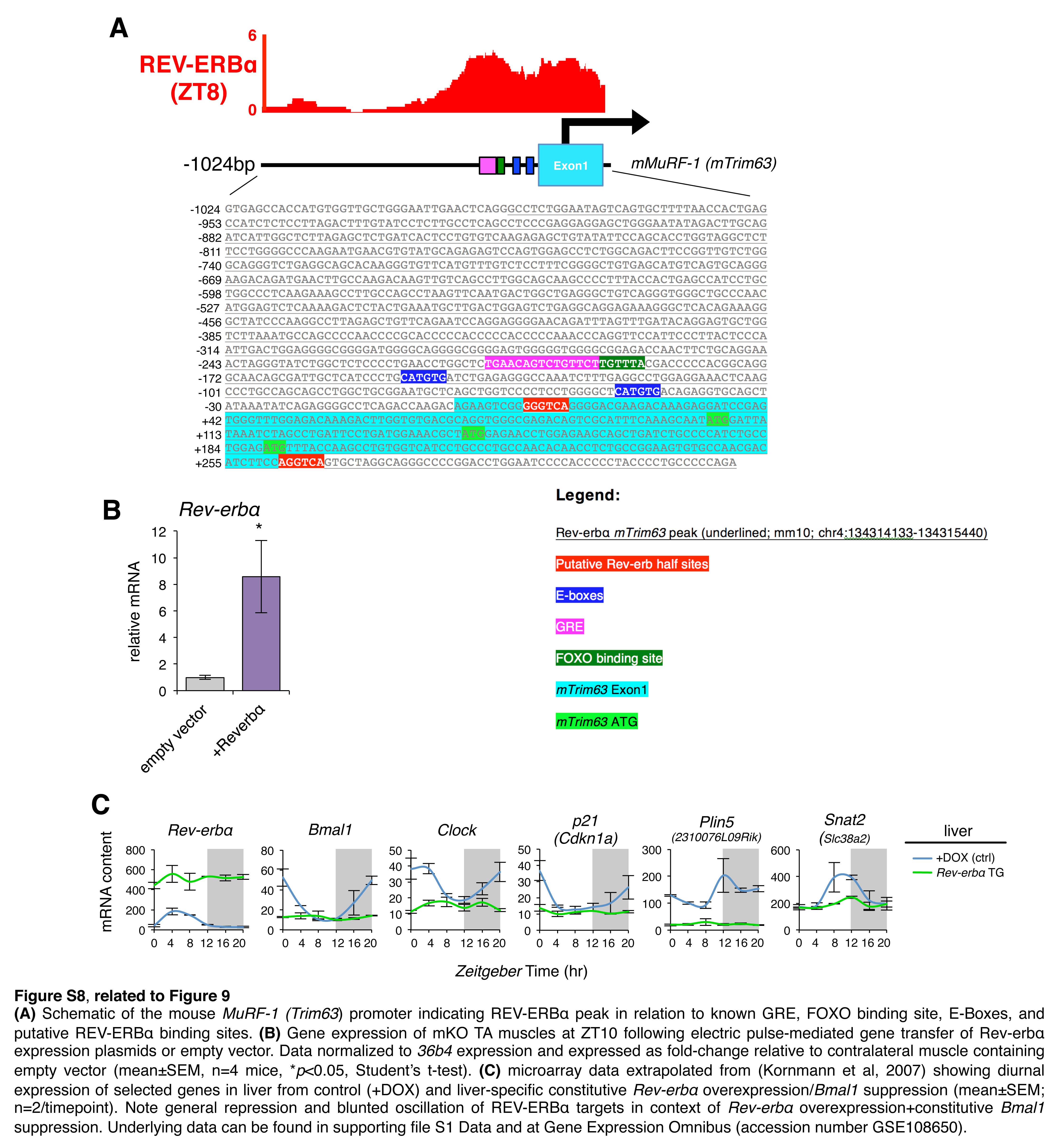

Supplement: S8 Fig — (TIF) [file pbio.2005886.s011.tif]
